# Revisiting Jonscher's universal power law: a modified formalism with Cole–Cole-type non-Debye dielectric relaxation for α-quartz-type GeO_2_

**DOI:** 10.1039/d6ra05511g

**Published:** 2026-08-20

**Authors:** Subrata Karmakar, Muralikrishna Patwari, Shreya Varsha Arun, Hitesh Borkar, Brahmananda Chakraborty

**Affiliations:** a Department of Physics, Manipal University Jaipur Jaipur Rajasthan 303007 India subrata.karmakar@jaipur.manipal.edu; b Chaitanya Bharathi Institute of Technology Osman Sagar Rd, Kokapet, Gandipet Hyderabad Telangana 500075 India; c Department of Physics, School of Physical Sciences, Amrita Vishwa Vidyapeetham Amritapuri Kerala-690525 India; d Department of Physics, NIT Warangal Telangana-506004 India; e High Pressure and Synchrotron Radiation Physics Division, Bhabha Atomic Research Centre Mumbai-400085 India brahma@barc.gov.in; f Homi Bhabha National Institute Anushaktinagar Mumbai-400094 India

## Abstract

The present work expounds the high-temperature dielectric response and conduction mechanism in ultrawide bandgap α-quartz-type GeO_2_ through a combined experimental and density functional theory (DFT) study. The Rietveld refinement of X-ray diffraction (XRD) analysis revealed the single-phase α-quartz-type GeO_2_ with high crystallinity, confirming phase purity and structural integrity. The distinctive lattice dynamics and bonding characteristics of the tetragonal α-quartz-type phase structure were further confirmed by its signature A_1g_ and E_g_ modes of Raman spectroscopy. The development of a dense microstructure with uniform grain growth in the sub-micrometer range and elemental composition (Ge and O) was supported by field-emission scanning electron microscopy (FESEM) and energy-dispersive X-ray spectroscopy (EDS). The UV-vis spectroscopic optical measurements revealed a broad experimental bandgap of ∼5.8 eV, in line with the electronic structure and density of states determined by DFT. The metal–oxide (M–O, O–M–O) and hydroxyl stretching (M–OH) vibrational modes of GeO_2_ are further reinforced by Fourier transform infrared (FTIR) spectroscopy. The dielectric permittivity (*ε*_r_) increased significantly from 7.2 to 33.5 between 573 K and 773 K, an effect attributed to defect-assisted processes and thermally activated space-charge polarization. The obtained Cole–Cole parameter (*α*) lies between 0.2 and 0.8, which recommends the non-Debye type dielectric relaxation process. The frequency-dependent ac conductivity followed a modified Jonscher's power law, 
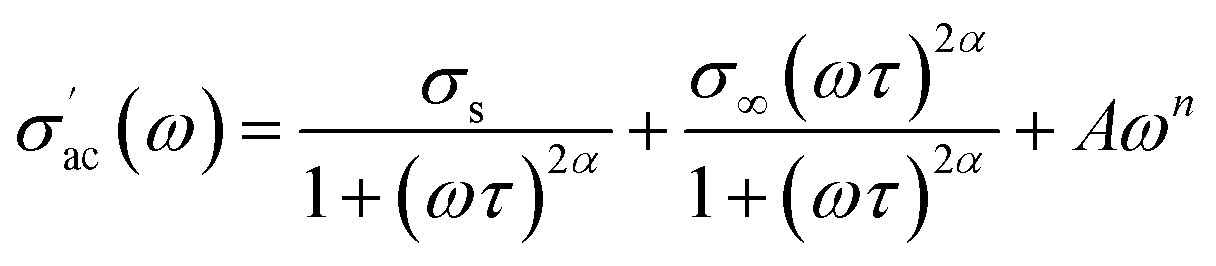
, and the variation of frequency exponent (*n*) with temperature indicates correlated barrier hopping conduction between localized states. The dc activation energy (*E*_dc_) was estimated to be ∼0.98 eV using the Arrhenius equation. The impedance and complementary modulus spectroscopy reveal non-Debye-type dielectric relaxation, negative temperature coefficient of resistance (NTCR), and activation energy (*E*_a_) for grain ∼0.48 eV and grain boundary conduction ∼0.81 eV, respectively. The density functional theory (DFT) calculations supported the experimentally reported polaronic transport behavior by providing a microscopic understanding of the density of states (DOS), theoretical dielectric constants, charge localization, and a theoretical band gap of ∼4.59 eV. This study provides important insights into the dielectric response, conduction kinetics, and electrical transport of tetragonal α-quartz-type GeO_2_, highlighting it as a promising candidate for next-generation high-temperature power electronics, dielectric devices, and harsh-environment functional systems.

## Abstract 2

The present work expounds the high-temperature dielectric response and conduction mechanism in ultrawide bandgap α-quartz-type GeO_2_ through a combined experimental and density functional theory (DFT) study.

## Graphical abstract

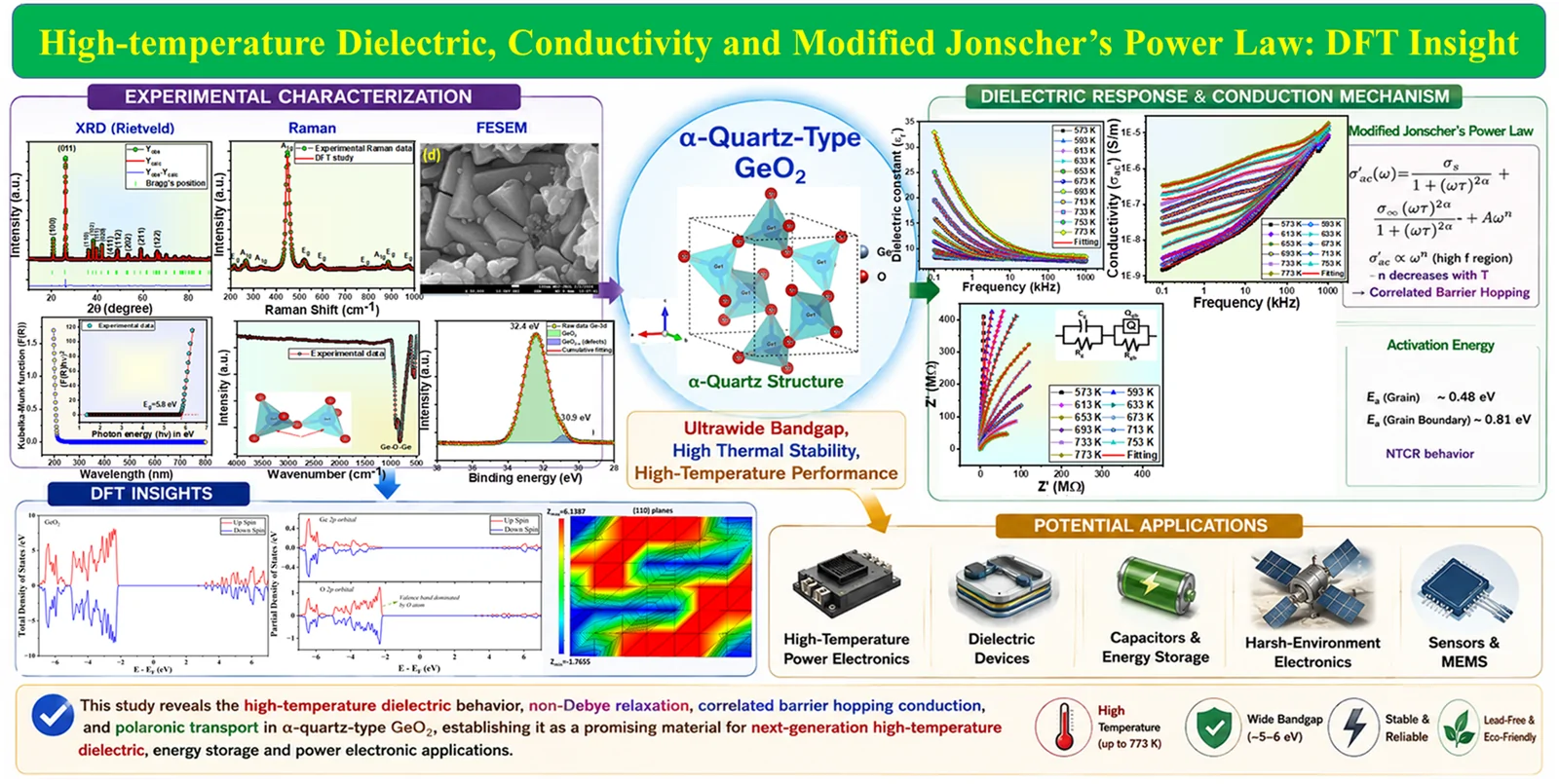

## 1 Introduction

Recently, ultrawide bandgap (UWBG) oxide materials have attracted considerable research interest due to their superior electrical insulation, high breakdown field strength, good thermal stability, and suitability for high-temperature electronic applications. Compared to traditional semiconductors like silicon (Si), germanium (Ge), and even wide bandgap materials like silicon carbide (SiC) and gallium nitride (GaN), the UWBG materials usually have a bandgap of greater than 3.4 eV, such as aluminum nitride (AlN), diamond, β-gallium oxide (β-Ga_2_O_3_), and α-quartz type germanium dioxide (GeO_2_).^1,2^ The high breakdown electric field in UWBGs enables their operation at elevated voltages with reduced power loss and enhanced efficiency, which makes them ideal for applications in power electronics, electric vehicles, renewable energy, and aerospace. Their excellent thermal stability allows reliable performance in extreme temperatures, making them suitable for harsh environments like space technology, defense systems, and high-temperature sensors. Their optical transparency in the UV region enables use in UV photodetectors and deep-UV optoelectronics. Furthermore, their strong dielectric strength and low leakage current enhance their applicability for gate dielectrics, MOS devices, and high-frequency communication systems.^3,4^

Among various UWBG oxides, quartz-type tetragonal α-quartz GeO_2_ is an ultra-wide-band-gap (UWBG) semiconductor that is appealing for next-generation power electronics, deep-UV optoelectronics, and transparent photonic devices due to its unique combination of wide bandgap, high mobility, superior thermal conductivity, high theoretical Baliga's figure of merit, and ambipolar doping potential. The α-quartz phase of GeO_2_ is very relevant for piezoelectric and optical applications because it possesses a tetrahedrally coordinated crystal structure, compared to the rutile GeO_2_.^5,6^ The unit cell crystal structure of amorphous, crystalline, and liquid α-GeO_2_, a chemical & structural analogue of SiO_2_ was explained by M. Micoulaut *et al.* by various spectroscopic techniques and a DFT study.^7^ The electronic structure and optical properties of amorphous GeO_2_, compared with those of amorphous SiO_2_, were investigated by B. Walker *et al.* using DFT calculations.^8^ S. Zhang *et al.* report the successful synthesis of ultra-long GeO_2_ nanowires by a facile and simple thermal evaporation process and observed their structural and optical properties.^9^ The GeO_2_ nanoparticle was synthesized *via* the hydrothermal method by M. Seal *et al.*, and its applications as electrically erasable memory and its photocatalytic properties were investigated.^10^ Recently, I. Rahaman *et al.* explored various aspects, such as structural, TEM, optical, and thermal conductivity of epitaxially grown GeO_2_ thin films by metal–organic chemical vapor deposition (MOCVD) techniques for advanced device applications.^10–12^ The impact of a GeO_2_ insertion layer between the Ru thin film bottom electrode and the SrTiO_3_ (STO) dielectric layer on the crystallization behavior and related electrical characteristics of the STO layer is examined by H. Paik *et al.*^13^ The dielectric response and ac conductivity study of (GeO_2_)_*x*_(SiO_2_)_1−*x*_ glasses prepared from aerogels was investigated by A. Feltri *et al.* for temperatures ranges 300–783 K in the frequency window 0.1 Hz to 100 kHz.^14^ Tingting Ji *et al.* presented an analysis of the dielectric characteristics and structural development of α-GeO_2_ during compression using first-principles calculations, alternating current impedance spectroscopy measurements, and *in situ* Raman spectroscopic observations.^15^ P. Papet *et al.* investigated that the α-GeO_2_ is highly promising for high-temperature piezoelectric applications and frequency-control devices due to its excellent thermal stability and elastic behavior up to 900 °C.^16^ Theoretically, first-principles calculations have demonstrated that α-quartz GeO_2_ exhibits significant structural flexibility, defect-tolerant surfaces, and advantageous dielectric properties, and its use as gate oxides or dielectric layers in ultrawide bandgap semiconductor devices is supported by those features of α-GeO_2_. Additionally, structural distortion and bond flexibility have a major impact on charge transport and polarization behavior according to computational studies, which is particularly crucial for dielectric and impedance research.^17,18^ These studies confirm that the quartz-type α-GeO_2_ is one of the most promising multifunctional materials for optical, dielectric, piezoelectric, and high-temperature electronic applications, and no studies on the high-temperature impedance, dielectric, and ac conductivity of α-quartz-type GeO_2_ have been reported so far. There are still very few systematic studies combining high-temperature dielectric relaxation, impedance spectroscopy, AC conductivity, electric modulus analysis, and DFT calculations for α-quartz-type GeO_2_, despite a extensive research on the structural and optical properties of GeO_2_. Therefore, we are motivated to investigate the high-temperature dielectric, impedance, modulus, and ac conductivity of α-quartz-type GeO_2_ using DFT analysis for its practical applications in next-generation UWBG semiconductor technologies. Most of the study on wide and ultrawide bandgap oxide materials examines the limitations of the conventional Jonscher's universal power law in analyzing the AC conductivity, highlighting its inability to address phenomena such as low-frequency plateau conductivity and non-Debye relaxation effects. The modified Jonscher power law combines the classical Jonscher power-law conductivity with Cole–Cole-type non-Debye dielectric relaxation, enabling the simultaneous description of high-frequency dispersive conduction, intermediate-frequency relaxation, and low-frequency electrode/interfacial polarization in a single analytical framework. This modification aims to enhance the understanding of thermally activated charge transport and establish a clearer connection between dielectric relaxation and electrical conduction mechanisms in α-quartz GeO_2_.

In this work, we have thoroughly examined the polaron-hopping conduction mechanism and high-temperature dielectric permittivity in quartz-type α-quartz-type GeO_2_ using a combined experimental and DFT approach. The XRD, Raman, and FTIR spectroscopy measurements were employed for structural confirmation, phase formation, and vibrational properties of GeO_2_. Surface morphology, microstructural features, and elemental compositions were investigated using FESEM and EDS analysis, while the optical bandgap was assessed using UV-vis spectroscopy. To elucidate the relaxation behavior and conduction dynamics, temperature-dependent impedance spectroscopy, dielectric measurements, electric modulus, and AC conductivity analysis were carried out in the frequency window 100 Hz to 1 MHz and temperature ranges from 573–773 K. Additionally, the intrinsic electronic structure and dielectric response of GeO_2_ were correlated with the experimental results using DFT simulations.

## 2 Experimental details

### 2.1 Materials & methods

High-purity germanium dioxide (GeO_2_, 99.99%) from Sigma-Aldrich was utilized as the precursor for synthesizing α-quartz-type GeO_2_, and deionized water was used for the entire experimental procedure. A controlled solid-state reaction method was used to create high-purity quartz-type GeO_2_. To obtain a fine and uniform powder, the analytical-grade GeO_2_ precursor was first thoroughly ground using an agate mortar, and later the powder was calcined in an air environment at a high temperature of 900 °C to promote phase transformation and stabilization of the quartz-type α-phase of GeO_2_. For dielectric measurements, the as-synthesized GeO_2_ powder with 3% polyvinyl alcohol (as a binder) was pressed into circular pellets of diameter ∼10 mm and thickness ∼1.5 mm using a uniaxial hydraulic press under a pressure of ∼5–7 tons. The pellets were subsequently sintered at high temperature at 950 °C to improve densification, reduce porosity, and enhance grain connectivity, which are essential for reliable electrical characterization. Prior to electrical measurements, both flat surfaces of the sintered pellets were polished to ensure parallel geometry and uniform thickness. The silver paste was applied on both sides of the pellet to make conductive electrodes, followed by drying at ∼150 °C to ensure good electrical contact and adhesion, forming a metal–insulator–metal (MIM) capacitor configuration.

### 2.2 Characterization techniques

The XRD measurement was performed using a Rigaku-made automated multipurpose X-ray diffractometer (model: SMARTLAB) to assess the phase purity, crystal structure, and lattice parameters of α-quartz-type GeO_2_, with diffraction patterns recorded over the 2*θ* range 15–85° using Cu-Kα radiation (*λ* ≈ 1.54 Å), step sizes 0.02°, and scan rates 5° min^−1^, respectively. The Raman-active phonon vibrational modes were investigated by the Horiba-made LabRAM HR Evolution Raman spectrometer using a 532 nm excitation laser source in the range between 200 and 1000 cm^−1^. The high-resolution FESEM images with EDS spectra & mappings were collected by a JEOL-made JSM-7610FPlus ultra-high-resolution electron microscope. Shimadzu UV-2600 UV-vis spectroscopy was used to examine the optical characteristics and bandgap of GeO_2_ in the wavelength range of 200–1000 nm. The functional groups and chemical bonds of GeO_2_ were determined using Bruker ALPHA FTIR spectroscopy in the mid-infrared range of 500–4000 cm^−1^.

High-temperature impedance, dielectric, and ac conductivity were examined by the HIOKI IM3570 impedance analyzer attached to a high-temperature measurement unit over a broad frequency window between 100 Hz and 1 MHz and broad high-temperature ranges 573–773 K. Each electrical measurement was conducted three times using three separately prepared pellet samples. The presented results had high experimental reproducibility and dependability, as evidenced by the observed standard deviation of the dielectric and conductivity values being less than 3–5%.

### 2.3 Computational details

The DFT calculations in this study were performed with the Vienna *ab initio* Simulation Package (VASP).^19–21^ The electron exchange and correlation effects were performed using the generalized gradient approximation in the Perdew–Burke–Ernzerhof (PBE) functional.^22^ The plane-wave basis was set at an energy cutoff of 520 eV. To account for interactions between the ionic cores and valence electrons, we relied on the projector augmented-wave (PAW) method. Atomic positions were relaxed until both the total energy and the interatomic forces reached convergence, with thresholds set at 10^−5^ eV and 0.01 eV Å^−1^, respectively. For Brillouin-zone sampling, a *Γ*-centered 6 × 6 × 5 *k*-point grid was used. The same computational setup was used to obtain the optical properties, which explains the trends observed in the experimental results. Since PBE is known to underestimate band gaps, we used the HSE06 hybrid functional instead to get a more accurate description of the electronic structure. This functional was used to generate the density of states of GeO_2_. Using VASPKIT, we post-processed the VASP output to obtain the real and imaginary parts of the dielectric function as well as the optical absorption coefficient. The nonlinear fitting procedures were executed in ‘Origin’ software using the fitting function builder, compiling, initial parameter selection, and convergence criteria (maximum iterations and fitting tolerance) with standard errors and 95% confidence intervals. While the experimental data were collected from real materials at finite-temperature conditions, the DFT calculations were carried out assuming an ideal, periodically ordered crystal structure at 0 K.

## 3 Results & discussion

**Fig. 1:**
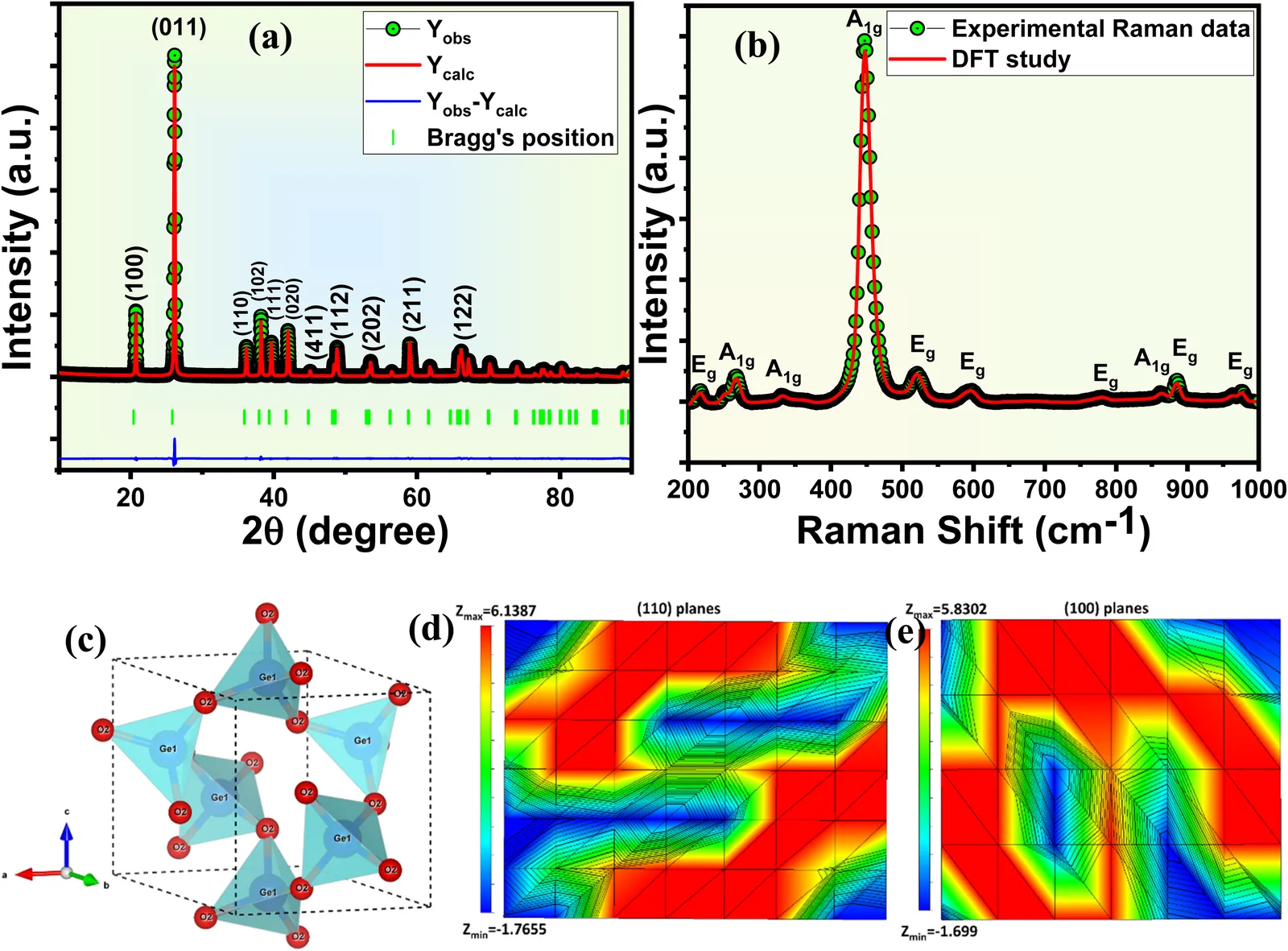
(a) The room-temperature XRD pattern with Rietveld refinement, (b) Raman spectra, (c) unit-cell crystal structure, (d and e) the MEM pattern along 110 and 100 planes of α-quartz-type GeO_2_.

The X-ray diffraction pattern of the α-quartz-type GeO_2_ in Fig. 1a indicates a well-crystallized quartz-type phase, with prominent peaks matching the hexagonal quartz structure. The absence of secondary phases confirms the high phase purity of the material. The trigonal crystal symmetry pertaining to the *P*3_1_21 (or *P*3_2_21) space group is confirmed by the Rietveld refinement of diffraction peaks observed at different characteristic 2*θ* positions, which correspond to planes like (100), (011), (110), (102), (111), (020), and so on.^23,24^ Good crystallinity and long-range structural ordering are indicated by the comparatively sharp and strong peaks from refinement with goodness-of-fit (*χ*^2^) ∼1.33, *R*_wp_ ∼ 7.37, and *R*_exp_ ∼ 6.36. The lattice parameters from refinement were obtained to be *a* = *b* = 5.08 Å, *c* = 5.72 Å, and *α* = *β* = 90°, *γ* = 120°, confirming the trigonal crystal framework.^25^ The Raman spectra of α-quartz-type GeO_2_ in Fig. 1b reveal several non-degenerate symmetrical stretching A_1g_ modes and doubly degenerate bending/stretching vibrational E_g_ modes, and further confirm the trigonal crystal symmetry. The A_1g_ modes in quartz-type α-quartz-type GeO_2_ originate due to the internal symmetric stretching of the Ge–O bonds, and E_g_ modes correspond to the asymmetric and bending distortions of the GeO_4_ tetrahedral unit.^26,27^ The low-frequency tiny peaks are associated with the lattice vibrations and framework motions. Fig. 1c depicts the crystal structure of GeO_2_, which is trigonal, similar to the symmetry of α-quartz, with each germanium (Ge) atom tetrahedrally coordinated to four oxygen atoms. Each GeO_4_ tetrahedron connects *via* shared oxygen atoms, creating a three-dimensional framework. The α-GeO_2_ exhibits better structural stability at high temperatures than α-quartz SiO_2_ (mostly used as dielectric layers), making it suitable for high-temperature dielectric applications. Its open framework and bond flexibility promote polarization under an electric field, thereby enhancing dielectric permittivity and relaxation behavior.^7,28,29^ The maximum entropy method (MEM) pattern was utilized to reconstruct the electron density distribution in GeO_2_ using XRD data that illustrate bonding characteristics and charge distribution. The MEM pattern along (110) and (100) planes of GeO_2_ is illustrated in Fig. 1d and e. The results show strong electron density around oxygen atoms and finite electron density along the bond path, indicating ionic character in Ge–O bonding and their strong covalent interaction, respectively. Additionally, it confirms the connectivity of the GeO_4_ tetrahedral network. The presence of low-density inter-tetrahedral regions indicates thermally activated hopping conduction, supporting its insulating nature and correlating with a stable dielectric response and high grain boundary resistance in impedance spectroscopy.^23,30^

**Fig. 2:**
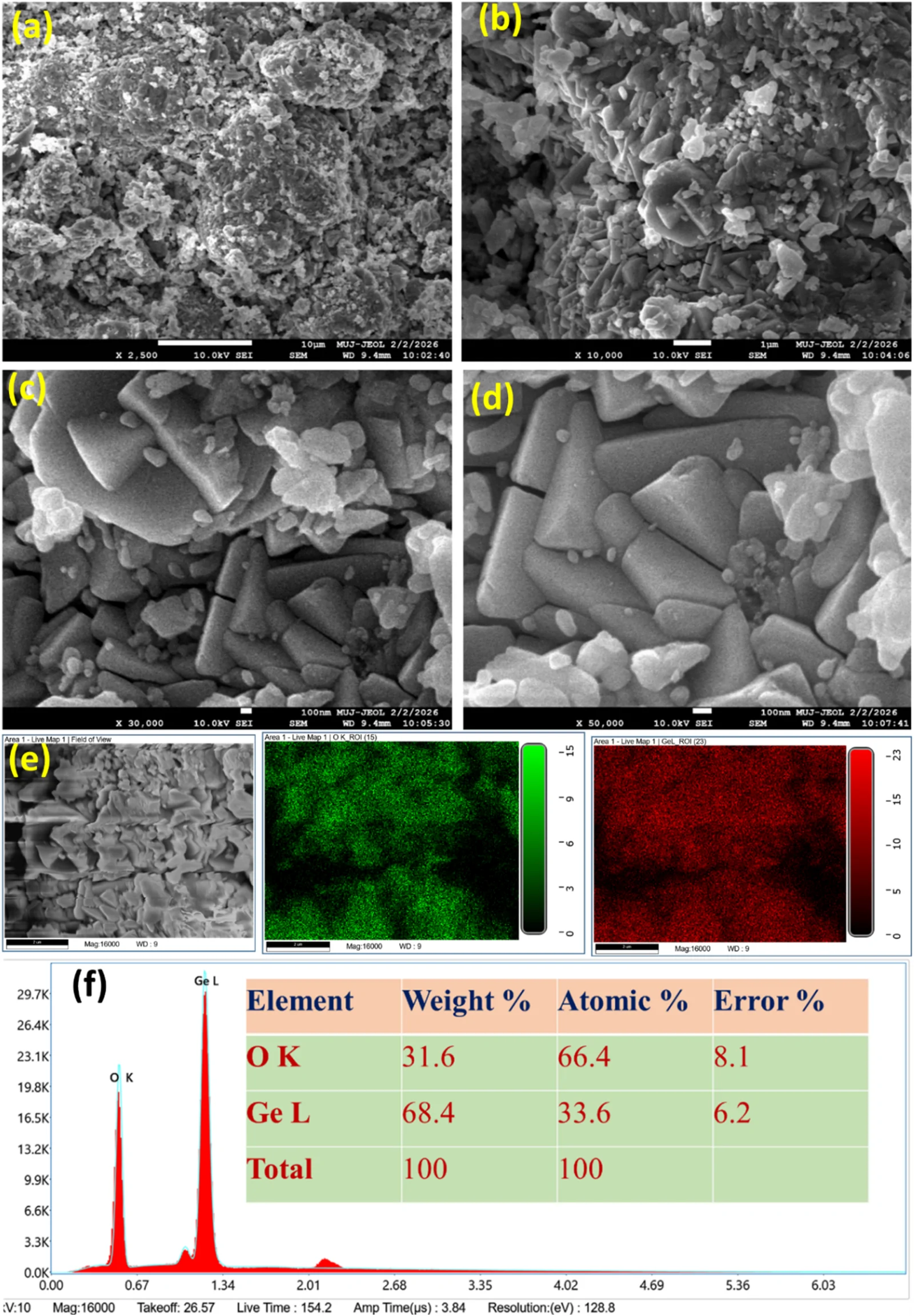
The high-resolution FESEM micrographs of GeO_2_ at different magnifications (a) 2500×, (b) 100 00×, (c) 300 00×, and (d) 500 00×, respectively. (e) EDS elemental mappings and (f) EDS spectra of GeO_2_.

The FESEM micrographs of α-quartz-type GeO_2_ are shown in Fig. 2a–d, which exhibit a densely packed polycrystalline morphology with distinct grains and grain boundaries. The higher-magnification images in Fig. 2c and d revealed closely connected grains with minimal voids and porosity, and effective grain growth during the sintering process. The crystallite sizes were obtained in the range between 300 nm and 800 nm, with an average value of 538.27 ± 0.23 nm. These closely spaced grains improve intergranular connection and lessen structural flaws, which are important factors in defining the electrical and dielectric characteristics of GeO_2_. Improved grain contact promotes effective charge transport across the bulk region, while decreased pore concentration reduces dielectric loss and charge carrier scattering. The electrical response is strongly influenced by barrier effects, as evidenced by the presence of discrete grain-boundary regions. The EDS mappings and spectra of GeO_2_ pellets are also illustrated in Fig. 2e and f, which demonstrate exceptional phase purity and homogeneity by confirming the stoichiometric composition of Ge and O with a uniform elemental distribution.

**Fig. 3:**
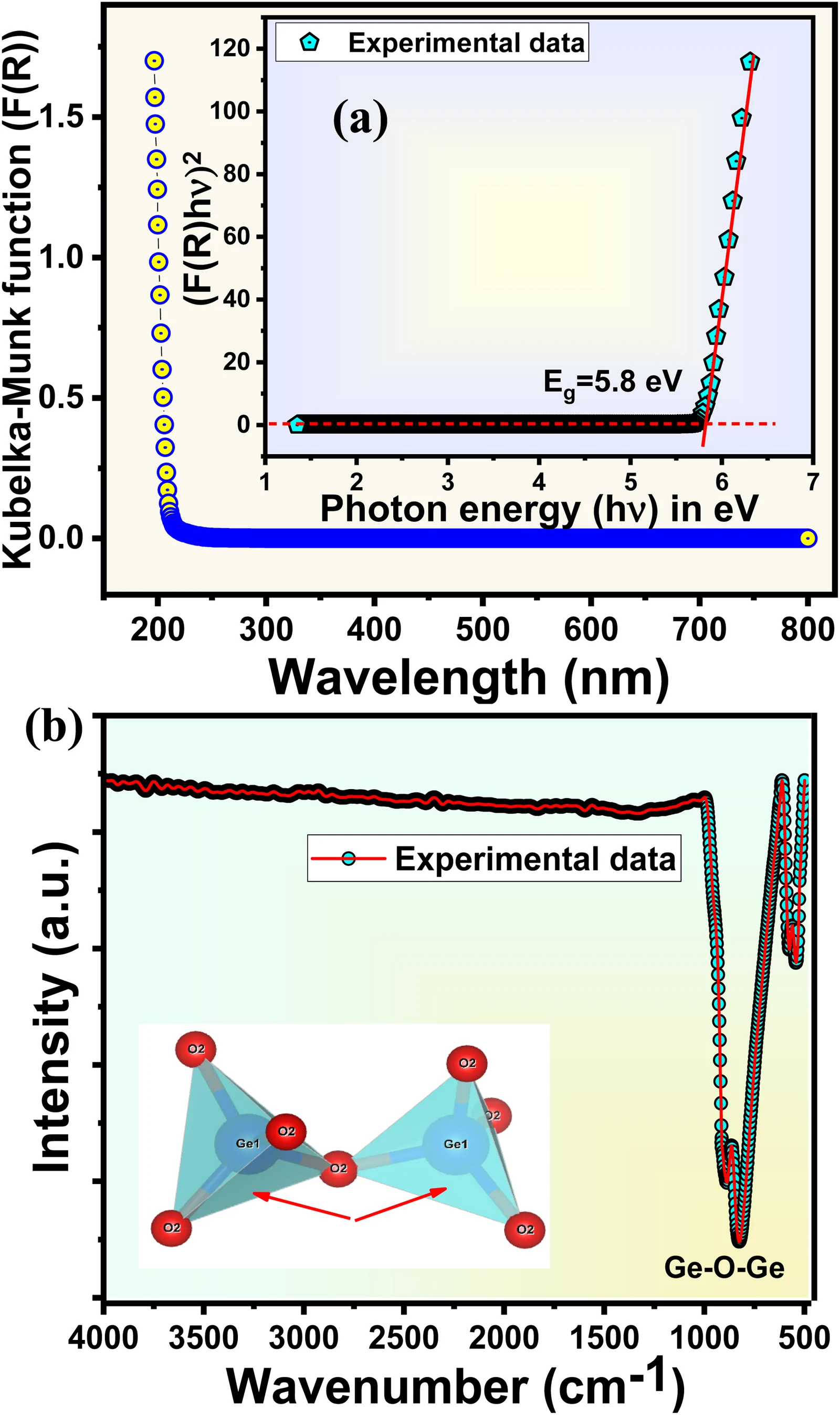
(a) The UV-vis absorbance spectra related to the Kubelka–Munk function *vs.* wavelength with bandgap, and (b) room-temperature FTIR spectrum of α-quartz-type GeO_2_.

The UV-visible diffuse reflectance spectroscopy (UV-DRS) analysis of quartz-type α-GeO_2_ reveals significant insights into its optical absorption properties and electronic structure. The absorbance spectra show a notable absorption in the ultraviolet range attributed to electronic transitions from the valence to the conduction band and a wide bandgap of α-quartz-type GeO_2_.^31^ The optical bandgap of α-quartz-type GeO_2_ was estimated from the Kubelka–Munk function (*F*(*R*)) and Tauc plot. The experimental diffuse reflectance spectra (*R*) were converted into an equivalent absorption coefficient using the Kubelka–Munk equation by,^32,33^1
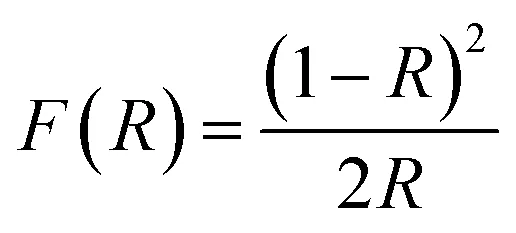
and its variation with wavelength (*λ*) is depicted in Fig. 3a. The bandgap was determined using Tauc's relation with the Kubelka–Munk function by,^34,35^

2 [*F*(*R*)*hv*]^2^ = *A*(*hv* − *E*_g_)

where *hv*, *A*, and *E*_g_ are the photon energy, proportionality constant, and optical bandgap, respectively. The proper transition model, based on linear fitting of the Tauc plot, as shown in the inset of Fig. 3a, is used to examine the band gap of GeO_2_. Strong Ge–O bonding and the tetrahedral GeO_4_ crystal framework are responsible for the observed wide band gap of ∼5.8 eV, which validates the ultrawide bandgap nature of quartz-type GeO_2_.^31,36^ The ultrawide bandgap nature of GeO_2_ promotes stable dielectric properties, high electrical resistance, and low leakage current density.

The FTIR spectra of α-quartz-type GeO_2_ in Fig. 3b reveal key insights into its bonding environment, lattice vibrations, and structural formation, and the observed absorption bands primarily stem from the vibrational modes of GeO_4_ tetrahedral units in the quartz-type crystal framework.^37,38^ The broad and intense downward doublet minima in the low wavenumber region at ∼536 cm^−1^ and ∼580 cm^−1^ correspond to symmetrical stretching of hexagonal GeO_2_. The strong absorption bands with peak doublets at ∼825 cm^−1^ and ∼897 cm^−1^ are attributed to the symmetrical and asymmetrical stretching of Ge–O–Ge linkages.^37^ The FTIR spectrum reinforces the successful formation of quartz-type GeO_2_ and the existence of strong Ge–O bonding within the tetrahedral framework.

**Fig. 4:**
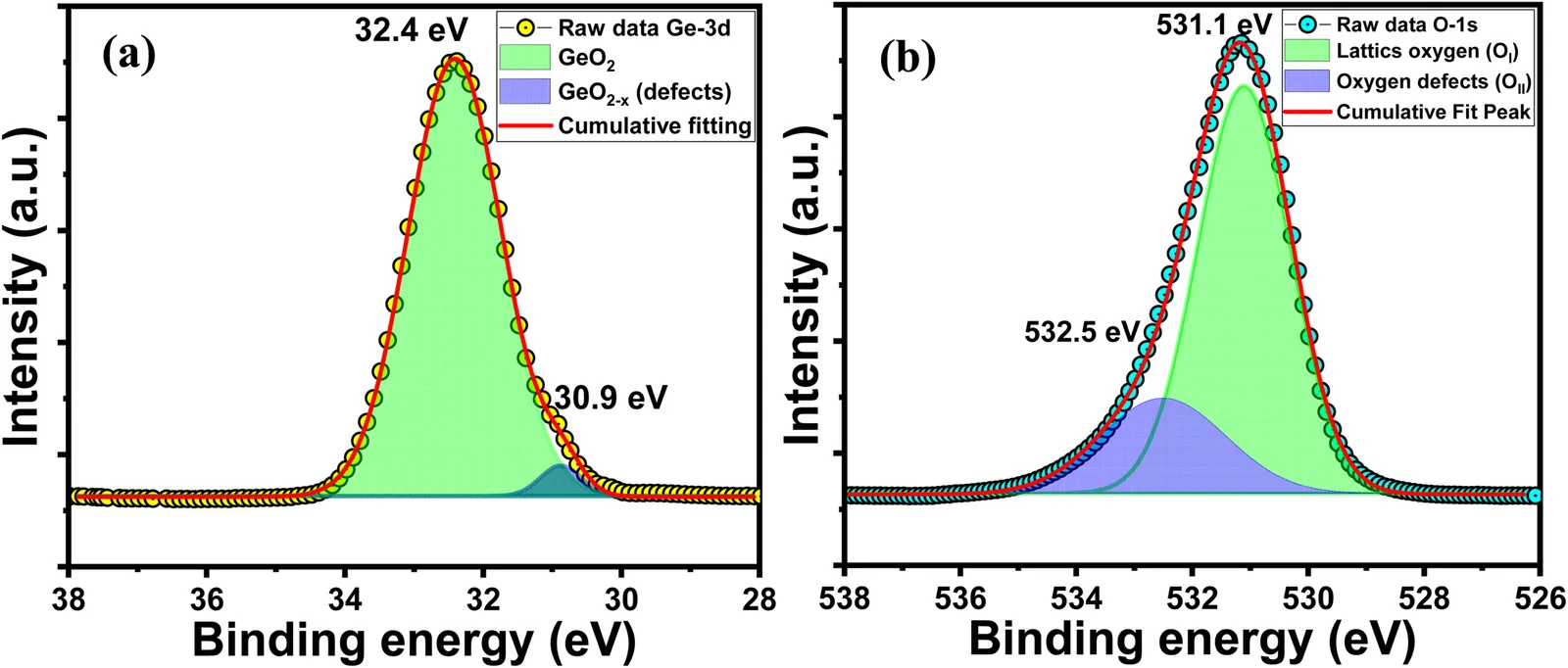
The individual scans of XPS spectra of (a) Ge-3d and (b) O-1s of α-quartz-type GeO_2_ with their deconvoluted peaks.

The chemical and oxidation states of the prepared GeO_2_ sample were examined using XPS analysis, and individual scans of Ge and O are shown in Fig. 4. The high-resolution Ge 3d core-level spectra in Fig. 4a exhibit a prominent broad peak at 32.4 eV, corresponding to the Ge^4+^ oxidation state, confirming fully oxidized germanium in stoichiometric GeO_2_. In addition to the main Ge^4+^ peak, a minor shoulder or secondary component was deconvoluted at a slightly lower binding energy of ∼30.9 eV, which appears due to the oxygen-deficient regions, near-stoichiometric germanium oxide (GeO_2−*x*_), or defect-associated Ge species.^39,40^ The O-1s core-level spectrum in Fig. 4b was deconvoluted into two components. The dominant broad peak at ∼531.1 eV is attributed to the lattice oxygen (O^2−^) coupled with Ge atoms in the GeO_2_ framework. The secondary shoulder peak, positioned at ∼532.5 eV, is associated with the oxygen-deficient defect states on the sample surface. The existence of such oxygen-related defects can have a substantial impact on the electrical and dielectric characteristics of GeO_2_ by promoting localized charge carrier hopping and interfacial polarization.^39–41^ The near-stoichiometric composition of GeO_2_ was confirmed by the atomic ratio of O/Ge, which was determined from the integrated peak areas after sensitivity factor correction, and it was found to be close to 1.942. To identify and quantify oxygen vacancies in detail, some more techniques like electron paramagnetic resonance (EPR) or positron annihilation spectroscopy (PAS) need to be performed.

**Fig. 5:**
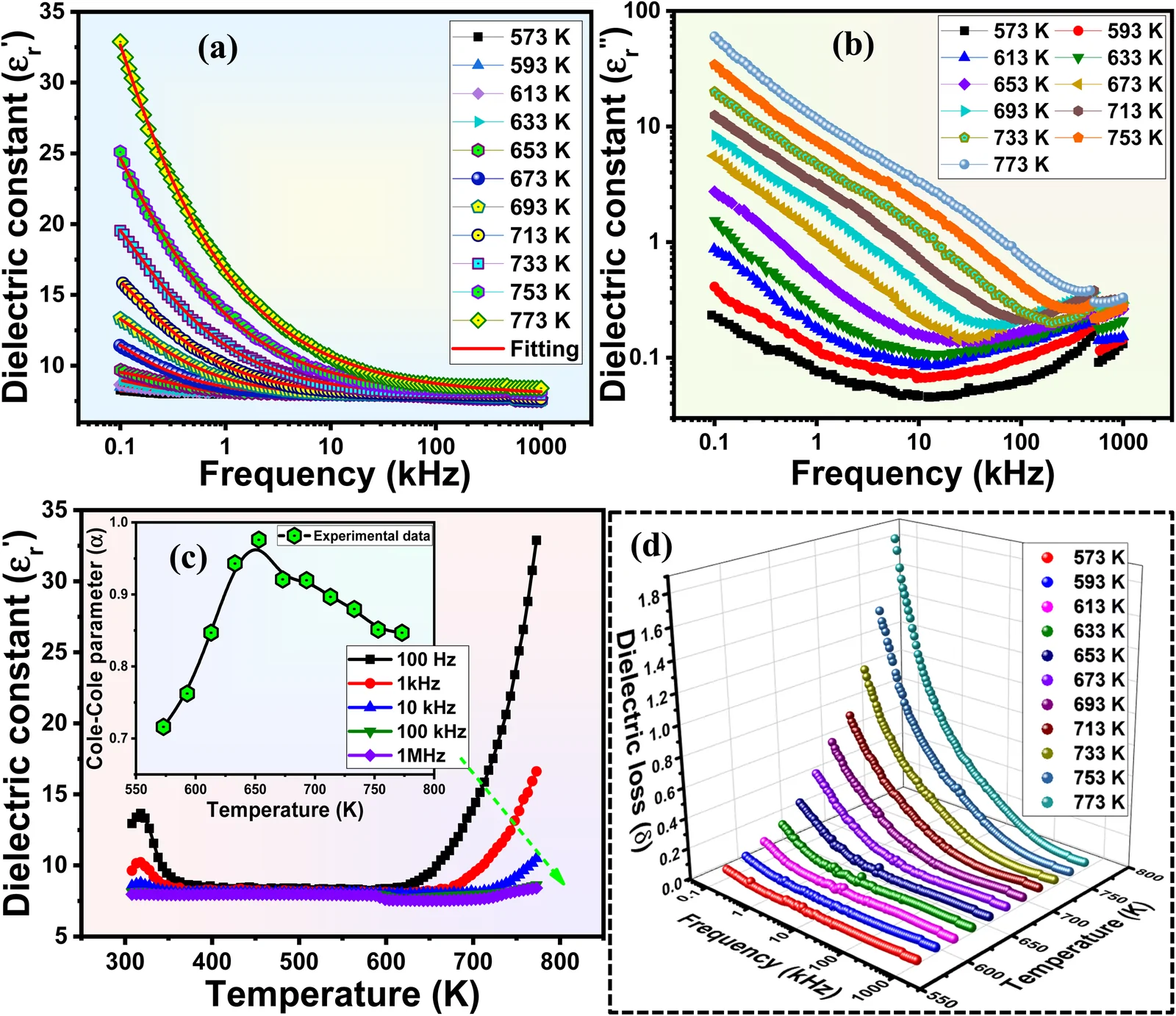
The frequency-dependent (a) real dielectric constant 
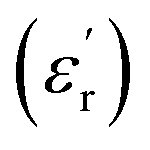
 and (b) imaginary dielectric constant 
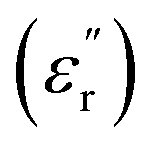
 of GeO_2_. (c)The dielectric constant 
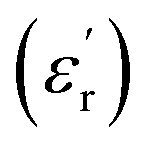
*vs.* temperature (K), (d) the 3D plot of dielectric loss (*δ*) with frequency and temperature of GeO_2_. The variation of the Cole–Cole parameter with temperature is shown in the inset of (c).

To comprehend the electrical polarization, dielectric relaxation, and charge transport mechanisms within the material, the dielectric response (dielectric constant and loss) of quartz-type GeO_2_ was examined as a function of temperature and frequency. The real 
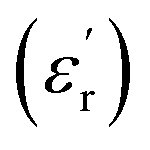
 and imaginary 
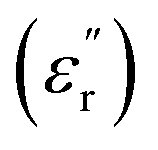
 dielectric constants were measured from experimental data using3
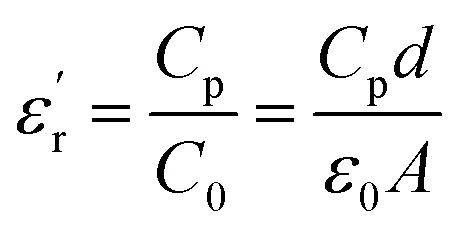
and,4
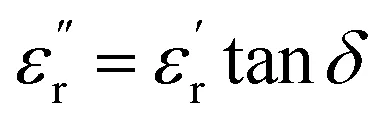
where, *d* and *A* are the thickness and area of parallel plate configurations, and tan *δ* is the dielectric loss tangent, respectively. The frequency dependence of the real dielectric constant 
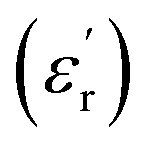
 is represented in Fig. 5a at various temperatures from 573 K to 773 K with Δ*T* = 20 K. The 
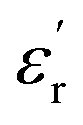
 value decreases with increasing frequency due to the progressive inability of various polarization mechanisms to follow the rapidly oscillating applied electric field. At low frequencies, all polarization types, mostly space-charge and interfacial polarizations, contribute to the dielectric response, as charge carriers accumulate at grain boundaries, defects, and interfaces, leading to a high dielectric constant. As frequency increases, heavier dipoles and charge carriers do not respond quickly enough to the alternating electric field, leading to a decrease in space charge and dipolar polarization, thereby reducing the dielectric constant.^42,43^ The dielectric constant 
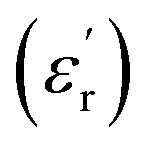
 follows the non-Debye type dielectric relaxation model governed by the Cole–Cole equation,^44–46^5
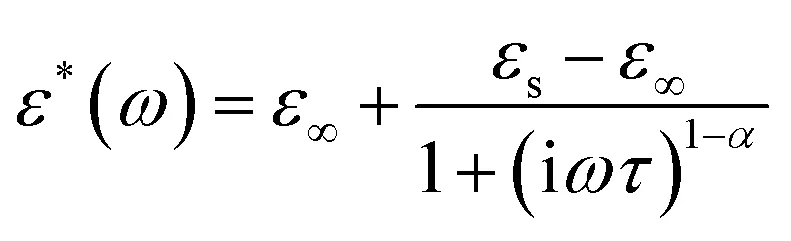
Separating the real and imaginary parts, we obtained,6
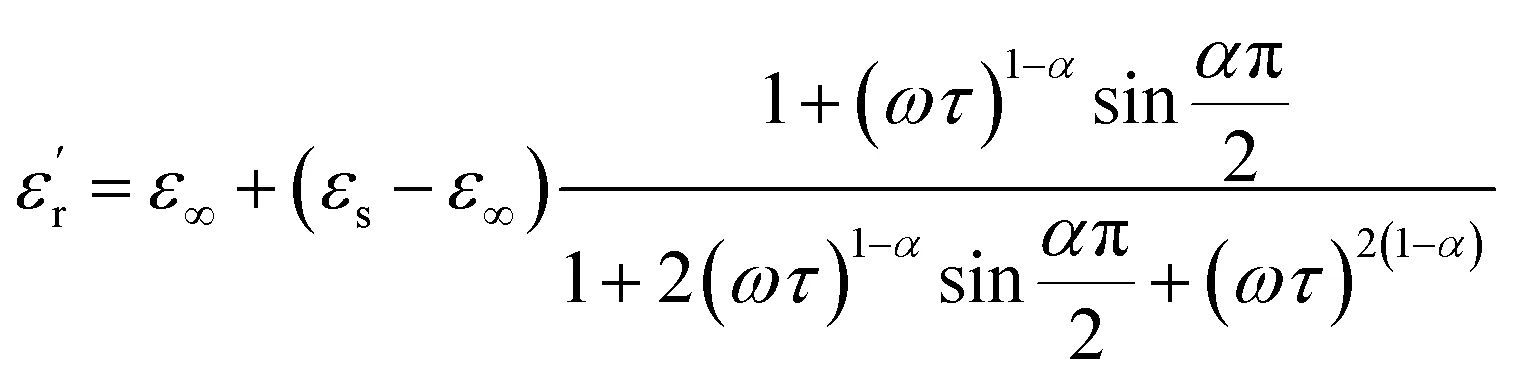
7
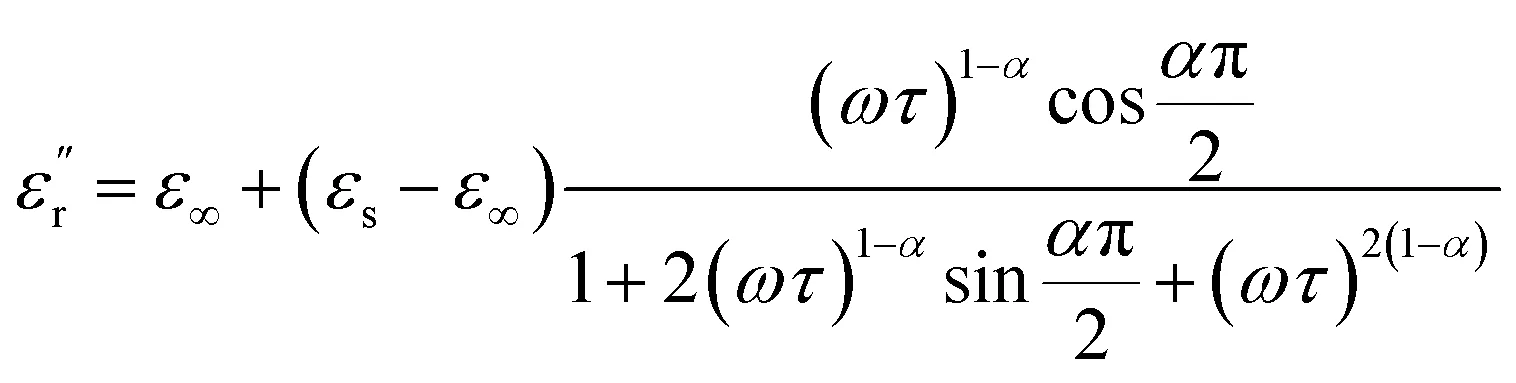
where, *ε*_∞_ and *ε*_s_ denote the dielectric constant at infinite and very low frequencies, respectively. The parameters *ω* (=2π*ν*), *τ*, and *α* represent the angular frequency, relaxation time, and Cole–Cole distribution parameter (ranging from 0 to 1). The non-Debye Cole–Cole model combined with Maxwell–Wagner interfacial polarization is generally the best fit for dielectric constant variations with frequency in GeO_2_ and similar oxide systems. Fig. 5a was modeled using eqn (6), yielding a Cole–Cole parameter (*α*) greater than 0 but less than 1, indicating a more stretched relaxation than Debye's. The imaginary component of the dielectric constant 
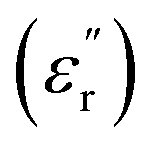
 relates to dielectric loss or energy dissipation in the material under an alternating electric field, as shown in Fig. 5b at different temperatures. Dielectric relaxation and charge-carrier hopping cause 
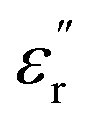
 to disperse with frequency, while space charge and interfacial polarization dominate the dielectric loss at low frequencies, resulting in high 
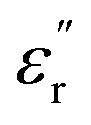
 values. Slight fluctuations and a gradual increase in 
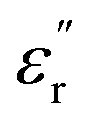
 at high frequencies may be due to electrode effects, resonance, measurement noise, or instrument limitations. The temperature dependence of the dielectric constant 
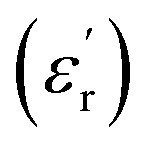
 of GeO_2_ across 303 K to 773 K is shown in Fig. 5c, where 
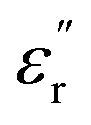
 exhibits minor fluctuations at low temperatures because of suppressed thermally activated polarization mechanisms, frozen dipoles, and reduced grain boundary effects. The low-frequency (100 Hz) dielectric constant of α-quartz-type GeO_2_ was approximately 10.5 at 303 K, increasing to about 33.1 at 773 K, aligning with prior studies.^17,47,48^ The degree of divergence from ideal Debye relaxation is represented by the Cole–Cole parameter (*α*), and its value less than unity suggests a dispersion of relaxation times due to localized defect states, grain-boundary heterogeneity, and structural disorder. The inset in Fig. 5c shows the Cole–Cole parameter (*α*), which ranges between 0.7 and 1 and decreases in the high-temperature region. This trend indicates a transition from non-Debye to nearly Debye-type relaxation as temperature rises. At lower temperatures, structural disorder and defect states cause a broad distribution of relaxation times, whereas higher charge and dipolar mobility with temperature result in more uniform relaxation, lowering *α*.^49^ The 3D graph of dielectric loss (*δ*) *versus* frequency and temperature is shown in Fig. 5d. The *δ* values decrease as frequency increases from 100 Hz to 1 MHz across 573 K to 773 K. At low frequencies (100 Hz to 10 kHz), *δ* is high (0.03–1.8), mainly due to Maxwell–Wagner interfacial and space charge polarization at different interfaces, causing substantial energy dissipation. As frequency increases, *δ* diminishes because dipoles and charge carriers lag behind the electric field. At higher frequencies (>10^4^ Hz), *δ* levels off at low values, indicating less energy loss and suitability for high-frequency uses. No distinct relaxation peaks are observed, suggesting that relaxation may occur outside the studied range or be broadly distributed. Additionally, *δ* rises with temperature at all frequencies, more noticeably at lower ones, as elevated thermal energy activates charge carriers, enhancing their hopping between localized states, increasing conductivity, and resulting in greater dielectric loss.^50^

The AC conductivity response of α-quartz-type GeO_2_ as a function of frequency and temperature provides important information on the dielectric relaxation processes and charge-transport mechanism. The real 
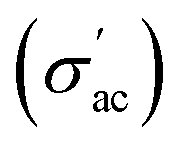
 and imaginary conductivity 
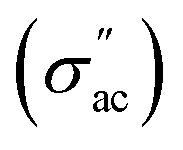
 were calculated from the impedance using the formula,^51,52^8
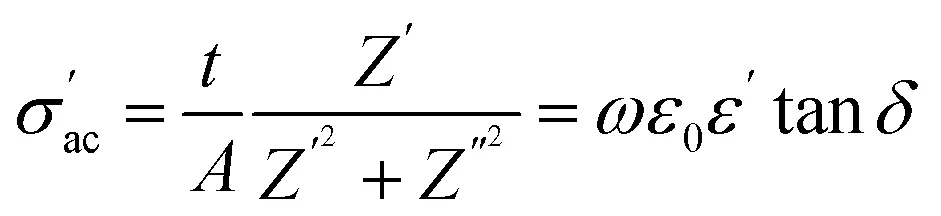
and,9
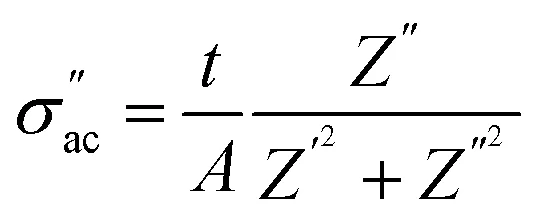
where *t* and *A* are the sample thickness and active electrode area, and *ω* and *ε*_0_ are the angular frequency and free-space permittivity, respectively. The AC conductivity in various ordered or disordered materials is described by the traditional Jonscher's universal power law as follows:10
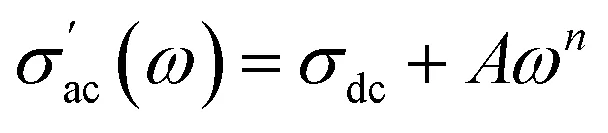


Although this empirical law effectively describes the dispersive behavior at high frequencies, it is frequently falls adequately describing systems that exhibit prominent dielectric relaxation processes (such as interfacial polarization or dipolar reorientation) in the intermediate and low frequency range, especially in wide or ultrawide bandgap semiconductor materials. To address this limitations we modified Jonscher's model by considering three independent assumptions-

(i) DC conductivity (*σ*_dc_ or *σ*_s_) (low frequency region).

(ii) Conductivity contribution from dielectric relaxation 
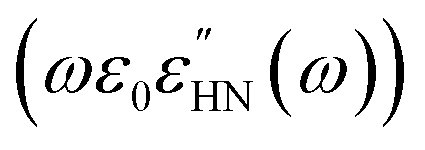
 (intermediate frequency region).

(iii) Dispersive hopping conductivity (*Aω*^*n*^) (high-frequency region).

The dispersive hopping conductivity from universal Jonscher's power law is associated with the charge transport that occurs through hopping/tunneling of localized charge carriers. The additional conductivity from dielectric relaxation arises due to dipolar reorientation, interfacial polarization (Maxwell–Wagner), or defect-related processes in the grain and grain boundary. Therefore, the real ac conductivity behavior was analyzed and fitted using the modified Jonscher power law combined with non-Debye-type Cole–Cole relaxation contributions,^53,54^11
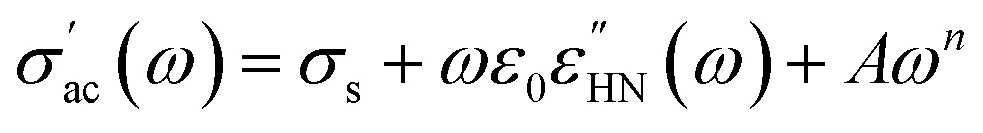
with,12
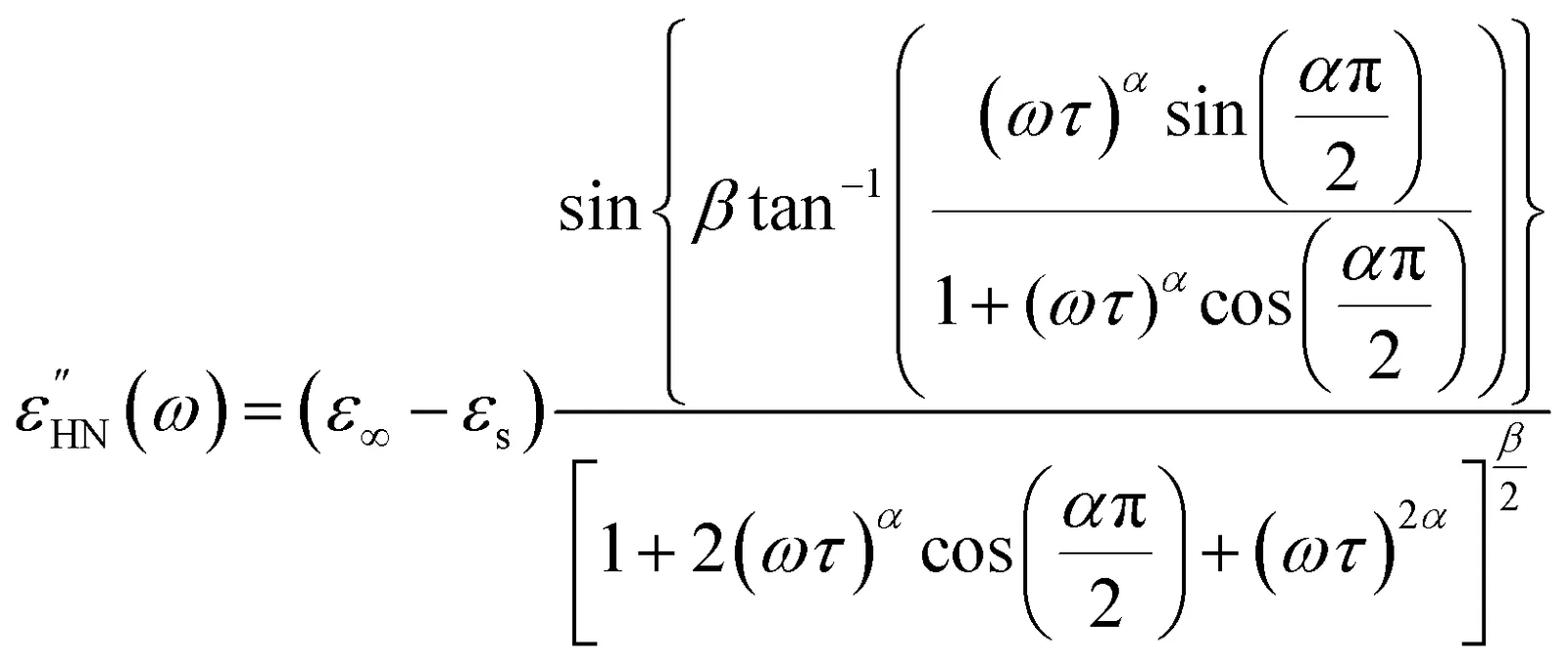


The parameters *α* (0 < *α* ≤ 1) and *β* (0 < *β* ≤ 1) are the Cole–Cole broadening parameter (symmetric broadening) and asymmetric broadening parameter, respectively. The Havriliak–Negami (HN) equation reduces to the Cole–Cole equation for *β* = 1. So, we have taken *β* = 1, and *α* < 1 for the non-Debye (symmetric broadening) relaxation model, and our 
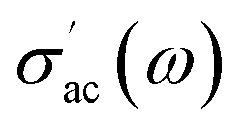
 spectra are best fitted by the modified equation,13
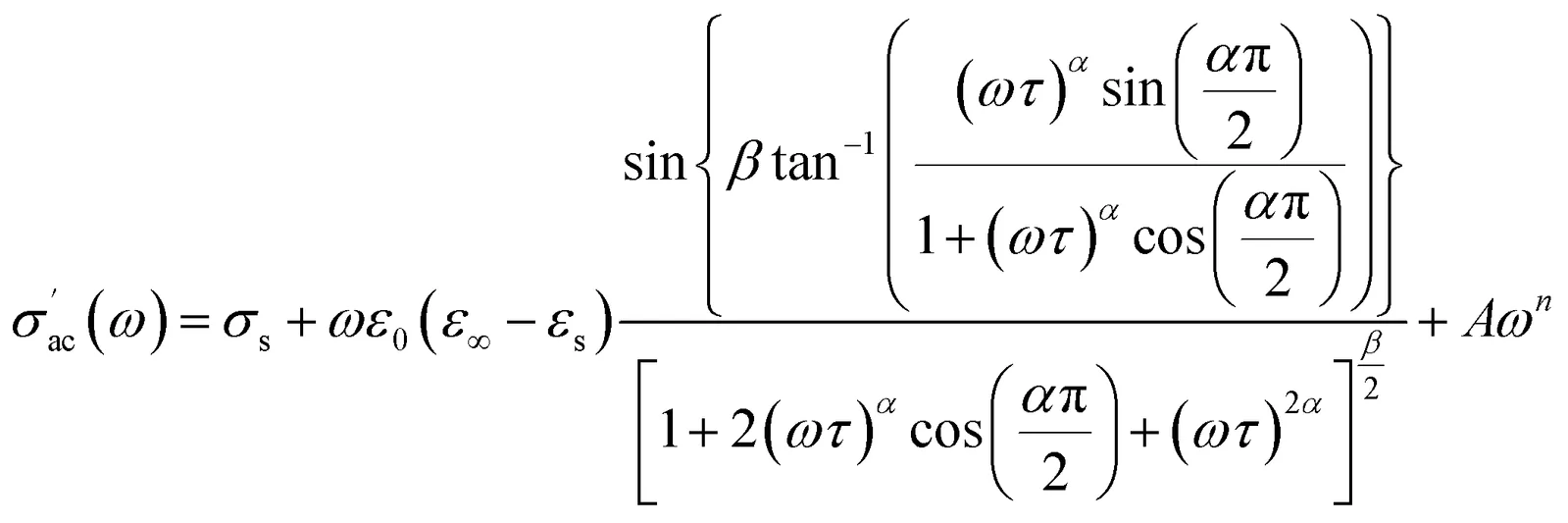
Taking *x* = (*ωτ*)^*α*^ and *β* = 1, we get14
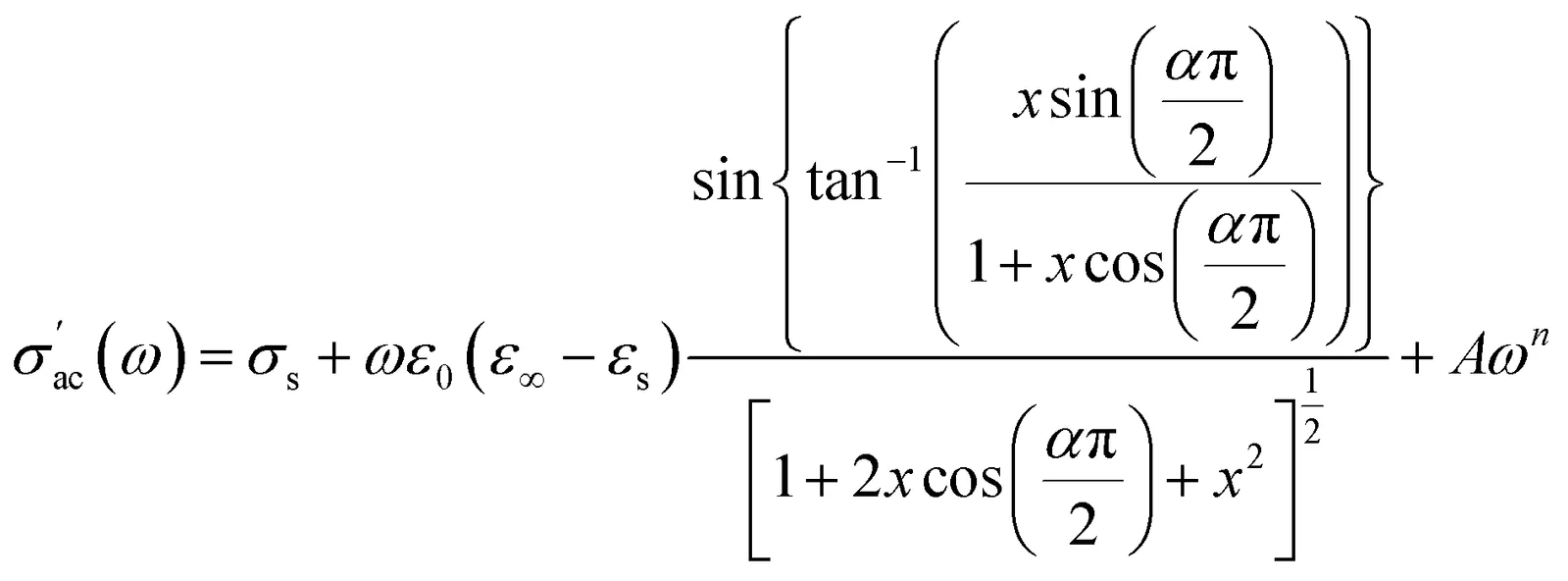
or,15
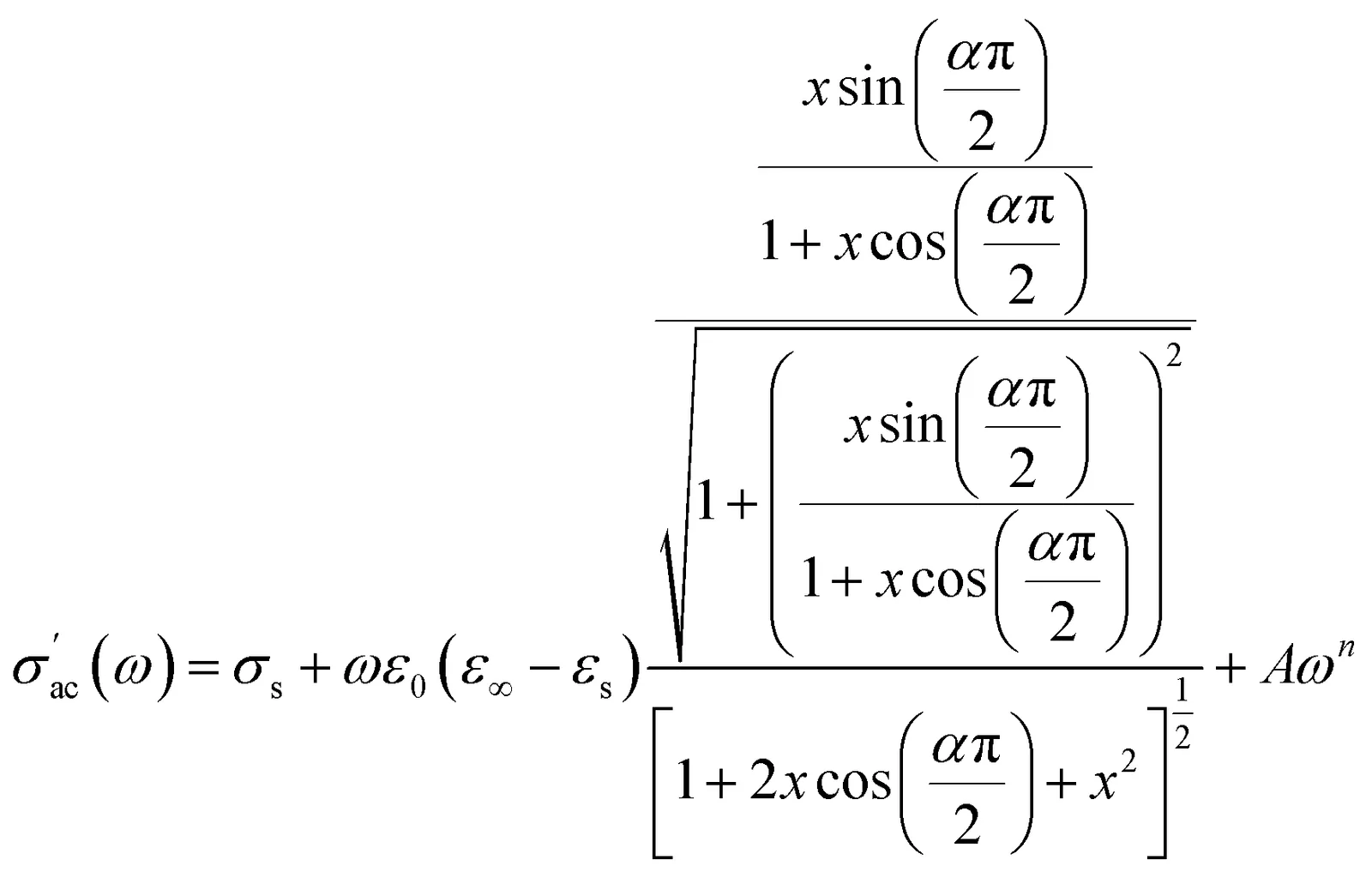
or,16
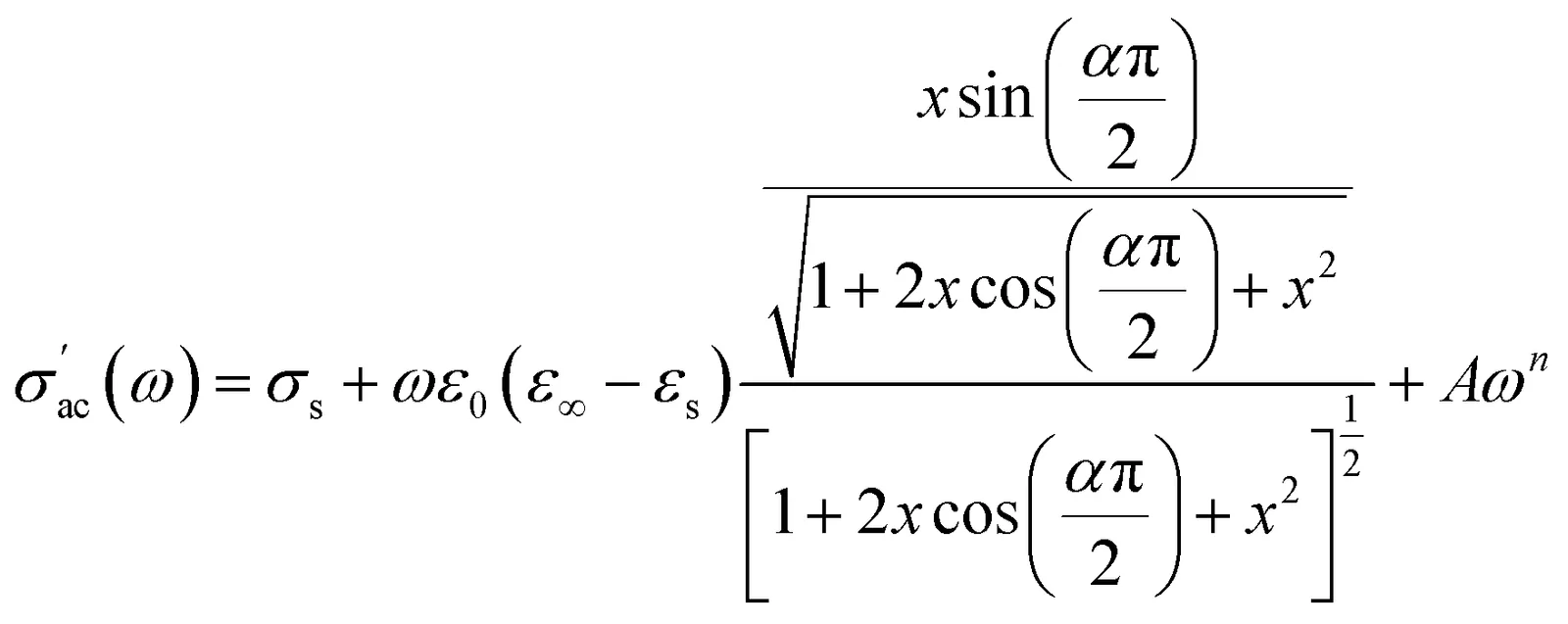
or,17
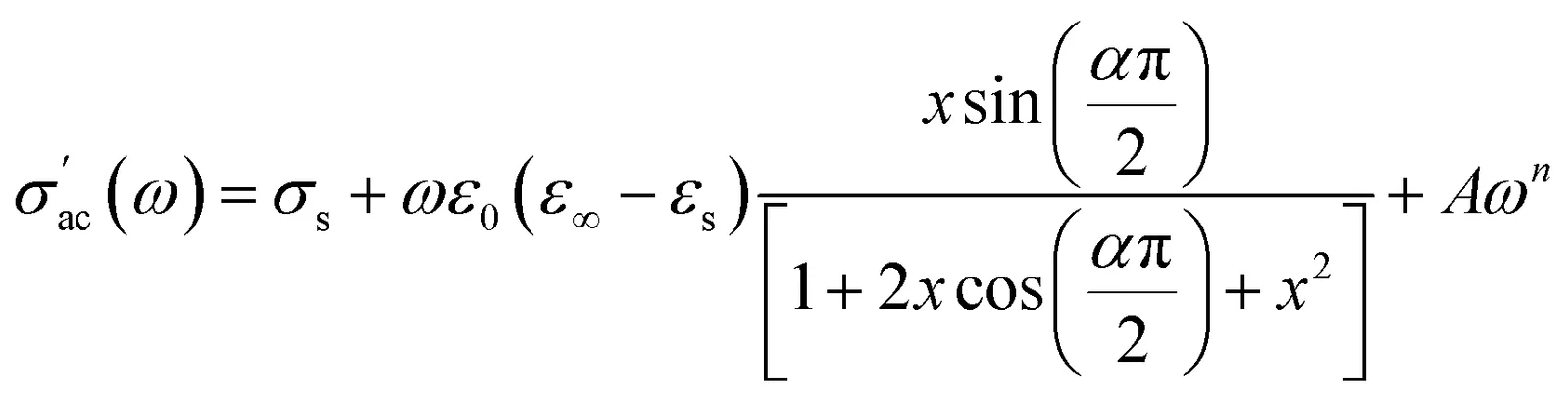
For most of the ceramic and ultrawide bandgap oxides, *α* ≈ 0.8–1.0, which is also obvious in our cases, and expanding18
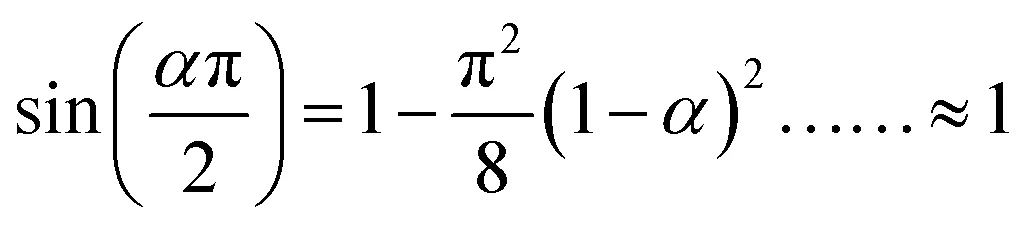
and19
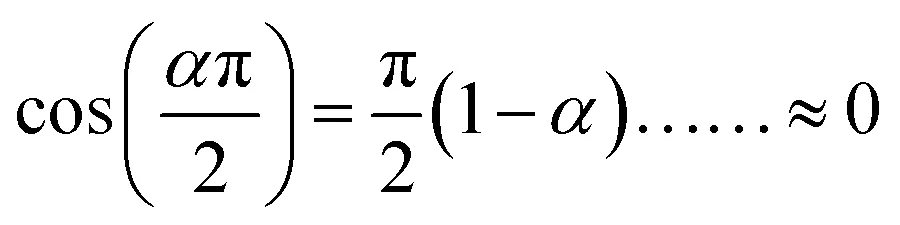
Putting the value of 
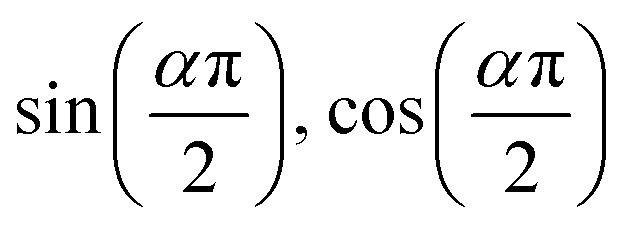
, and *x* in eqn (17),20
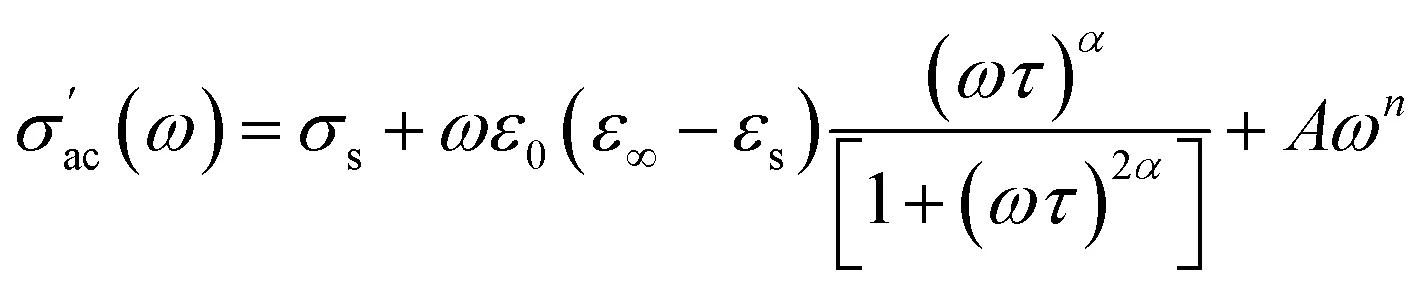


Substituting 
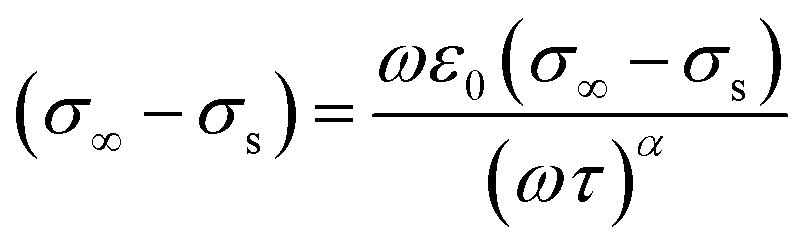
 (for non-Debye Cole–Cole relaxation), we can get21
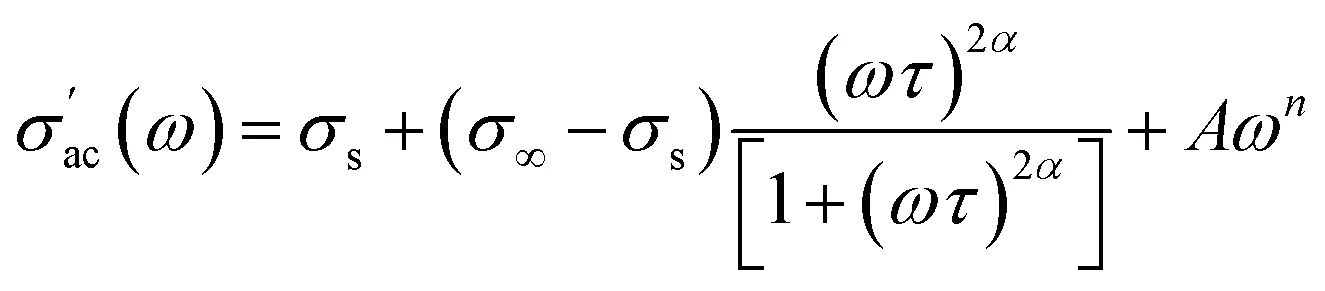
22
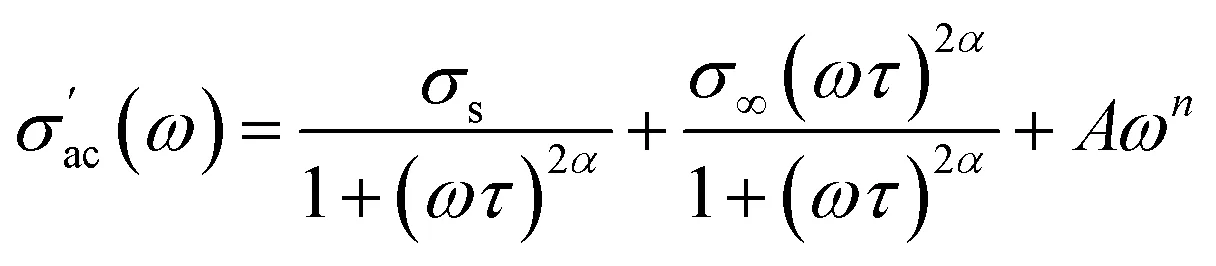
where, *σ*_s_ and *σ*_∞_ are the low-frequency static or dc conductivity and high-frequency limiting conductivity, *τ* and *A* are the characteristic relaxation time and temperature-dependent pre-factor constant, respectively. The relaxation time (*τ*) corresponds to the characteristic response time of dipoles or localized charge carriers which reduces as the temperature rises because of increased thermally triggered hopping. The term *n* is the frequency exponent that describes the degree of interaction between mobile charge carriers and the lattice and provides detailed information about the conduction mechanism governing AC electrical transport. At the very low frequency limit 
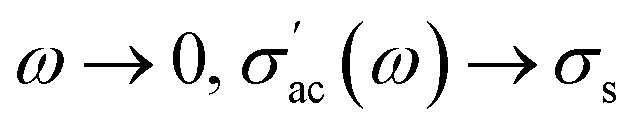
, and at the high frequency limit, 
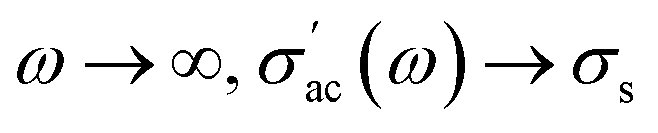
. When *α* = 1 and *β* = 1 for the ideal Debye-like relaxation model, the above eqn (22) for ac conductivity becomes,23
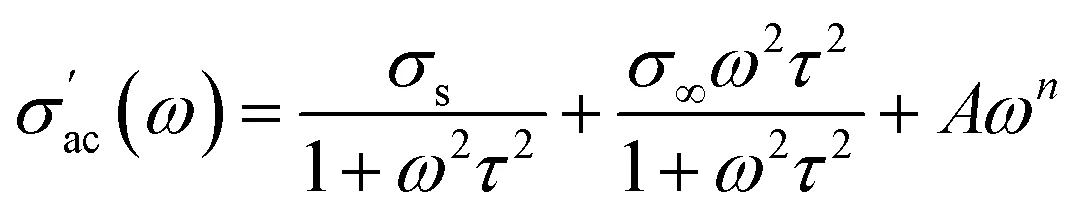


**Fig. 6:**
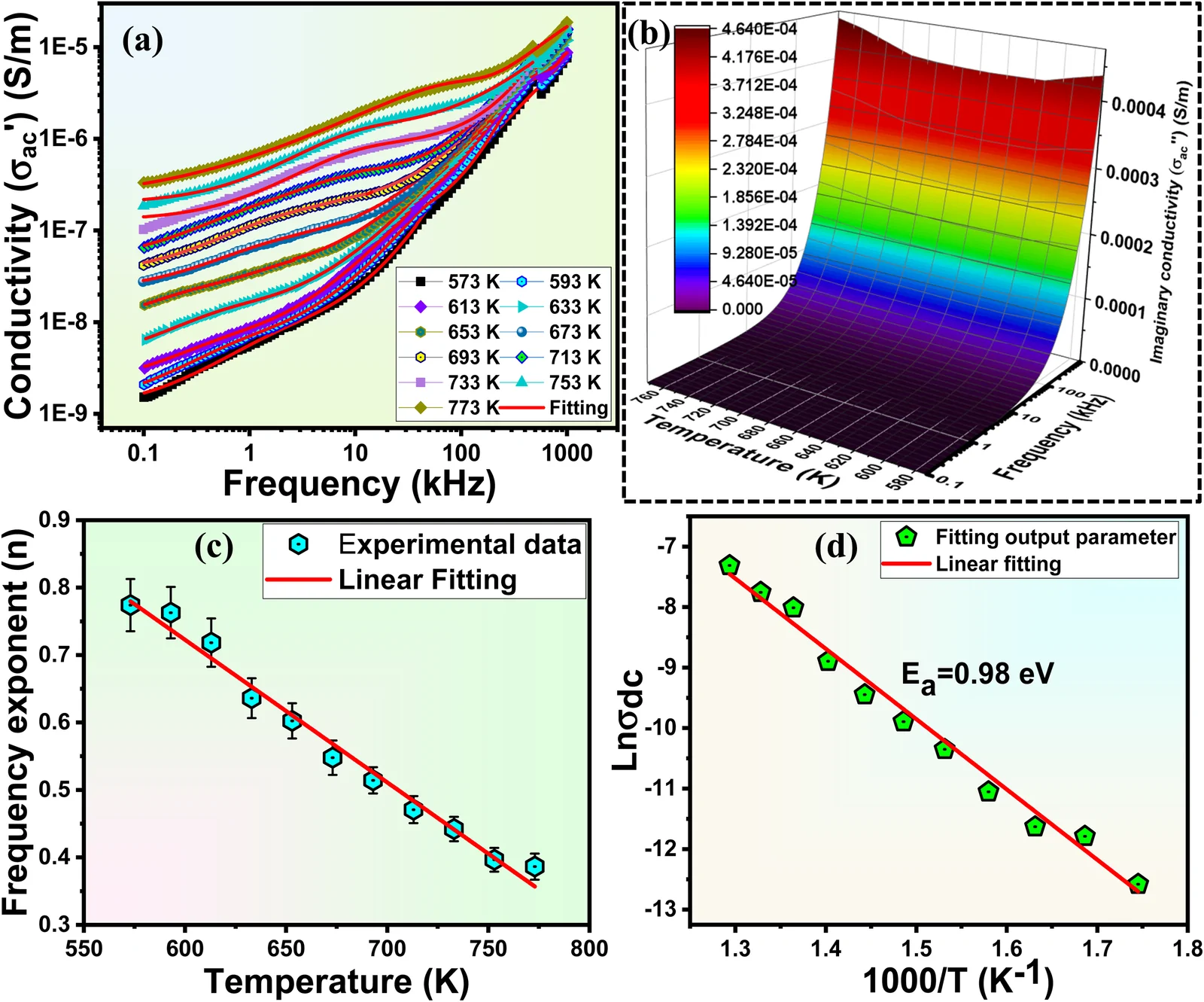
(a) The real part of ac conductivity 
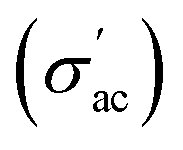
 with frequency, (b) the 3D plot of imaginary ac conductivity 
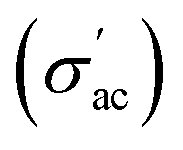
 with frequency and temperature of GeO_2_. (c) The frequency exponent (*n*) with temperature (K), (d) the Arrhenius plot of ln(*σ*_dc_) *vs.* 1000/*T* (K) for the calculation of activation energy.

Several previous works follow eqn (23) to fit their ac conductivity data,^55–59^ but our experimental ac conductivity of GeO_2_ is best fitted by non-Debye relaxation-modified ac conductivity of eqn (22) using the *α* values obtained from dielectric measurements. This phenomenological approach is mostly useful for materials that show thermally triggered hopping conduction and non-Debye dielectric relaxation. The frequency dependence of the ac conductivity 
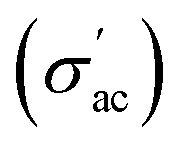
 of α-quartz-type GeO_2_ is shown in Fig. 6a at various temperatures from 573 K to 773 K. The 3D plots of imaginary conductivity 
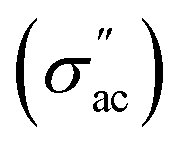
 as a function of frequency and temperature are also shown in Fig. 6b for all aforementioned temperatures. The AC conductivity increases with both temperature and frequency, suggesting that the material exhibits a thermally triggered charge transport process. At higher frequencies, increased AC conductivity is the result of localized charge carriers hopping between defect states and localized trapping centers. The semiconducting nature of GeO_2_ is confirmed by the temperature-dependent increase in conductivity, which also implies that thermally activated hopping mechanisms are responsible for effective charge transfer *via* oxygen vacancies, grain-boundary effects, and localized defect-induced polarization processes. The temperature dependence of the frequency exponent (*n*), derived from modified Jonscher's power law, was used to examine the conduction process in GeO_2_. The major conduction mechanism and the degree of carrier localization are reflected in the conductivity exponent (*n*) derived from the modified Jonscher model, and its temperature dependence is consistent with thermally stimulated hopping conduction, suggesting that charge transfer occurs *via* defect centers and localized states associated with mixed-valence ions. The observed *n* value in Fig. 6c decreases with increasing temperature, suggesting that the AC conduction process is mainly governed by the correlated barrier hopping (CBH) model. This model suggests that localized charge carriers are thermally activated and hop between defect states over potential barriers to facilitate charge transport. The frequency exponent (*n*) is related to the maximum barrier height (*W*_m_) by,^60,61^24
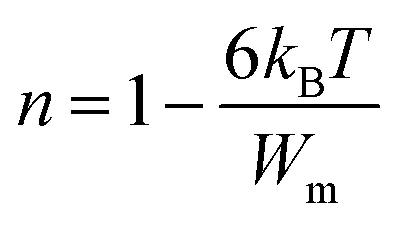
where, *k*_B_ is the Boltzmann constant (8.62 × 10^−5^ eV K^−1^). The maximum barrier height (*W*_m_) value of GeO_2_ was obtained at ∼0.28 eV from the slope of the *n vs. T* plot in the temperature range between 573 K and 773 K. The *W*_m_ < 0.5 eV indicates easy hopping of charge carriers between defect states or trapping centers and high defect-assisted conduction at high temperatures. The activation energy (*E*_a_) for dc conductivity was determined using the Arrhenius equation,^45,62^25
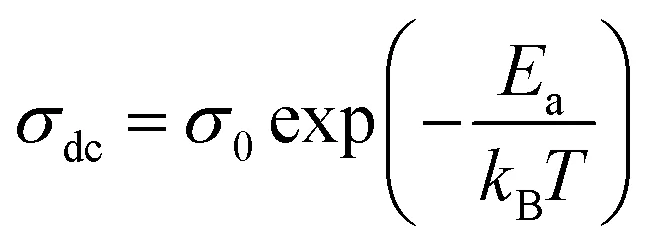
where *σ*_0_ is the pre-exponential factor. The ln *σ*_dc_*vs.* 1000/*T* (K) is shown in Fig. 6d, and the *E*_a_ value was calculated to be ∼0.98 eV for α-quartz-type GeO_2_. The activation energy values suggest thermally activated charge transport in GeO_2_ or GeO_2_-based oxide systems, driven by oxygen vacancies, localized defect states, and grain boundary effects. The intrinsic ionic/electronic conduction of Ge^4+^ and O^2−^ is low at low temperatures, but it conducts significantly with oxygen vacancies/defects at high temperatures above 573 K.

**Fig. 7:**
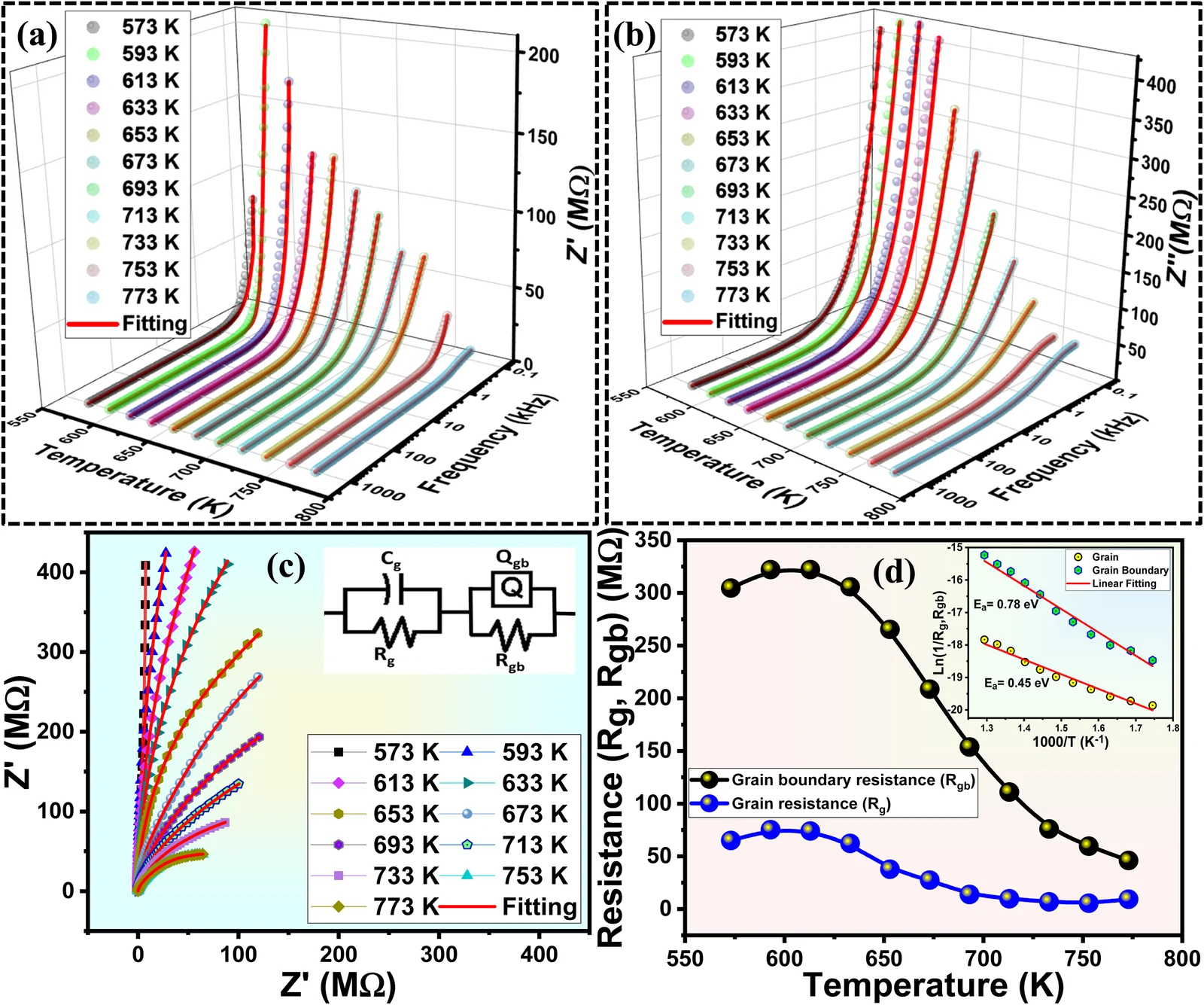
The 3D plots of (a) real impedance (*Z*′), (b) imaginary impedance (*Z*″) with frequency and temperature, (c) the Nyquist plot (*Z*′ *vs. Z*″) at different temperatures of α-quartz-type GeO_2_. (d) The variations of grain (*R*_g_) and grain boundary resistance (*R*_gb_) with temperatures and their activation energy (*E*_a_) in the inset of (d).

The real (*Z*′) and imaginary (*Z*″) impedance provide significant information about the dielectric relaxation and conduction mechanisms in α-quartz-type GeO_2_, especially at high temperatures. The *Z*′ and *Z*″ were calculated using the formulas *Z*′ = *Z* cos *θ* and *Z*″ = *Z* sin *θ*, where *θ* is the phase angle. The frequency dependence *Z*′ and *Z*″ at various temperatures from 573 K to 773 K is delineated in Fig. 7a and b, respectively. Both *Z*′ and *Z*″ decrease with increasing frequency and temperature. At low frequencies, both impedances are high due to grain boundary effects, electrode polarization, and localized charge accumulation, and it drops as the charge carriers gain energy to respond to the alternating electric field, improving electrical conduction. The decrease in *Z*′ with temperature indicates negative temperature coefficient of resistance (NTCR) behavior, confirming the semiconducting nature of the α-quartz-type GeO_2_ sample. At higher temperatures, thermally activated charge carriers more easily overcome grain boundary barriers, leading to lower resistive impedance. The Nyquist plots (*Z*′ *vs. Z*″) in Fig. 7c for temperatures 573 K to 773 K are fitted by (*R*_g_*C*_g_)(*R*_gb_*Q*_gb_) circuit network using ZSimpWin software, where the first block is a parallel resistor-capacitor circuit, representing the grain interior and the second block is a parallel circuit of a resistor and a constant phase element (CPE), representing the grain boundary contributions. The constant phase element (CPE) exponent describes the non-ideal capacitive behavior resulting from a distribution of relaxation times induced by microstructural heterogeneity.

Therefore, the *Z*′ and *Z*″ spectra in Fig. 7a and b are fitted by considering the equation,^63,64^26
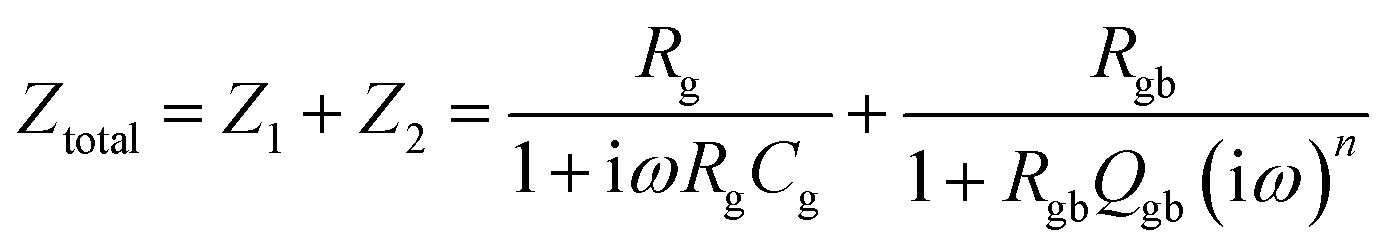
and,27
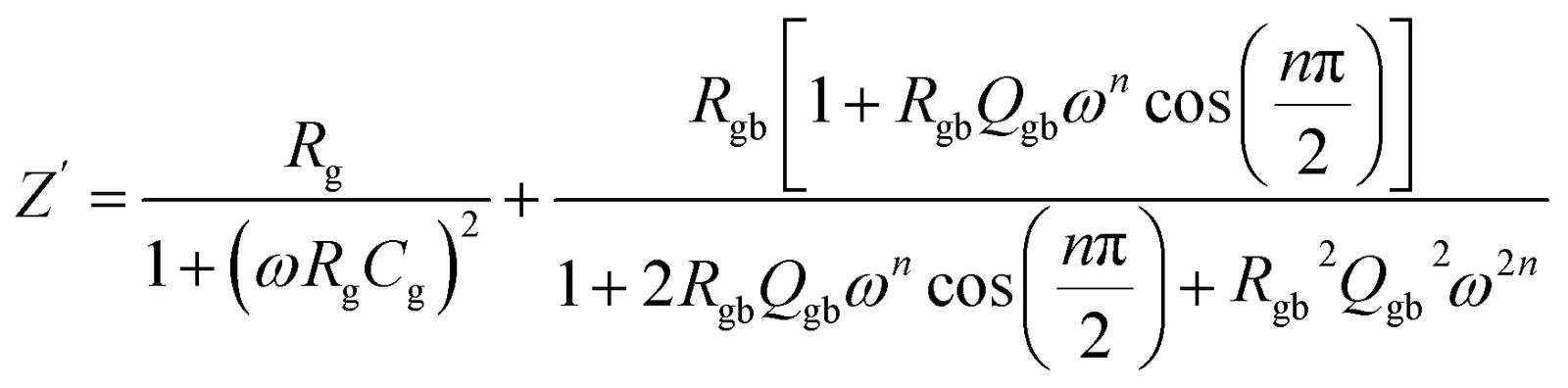
28
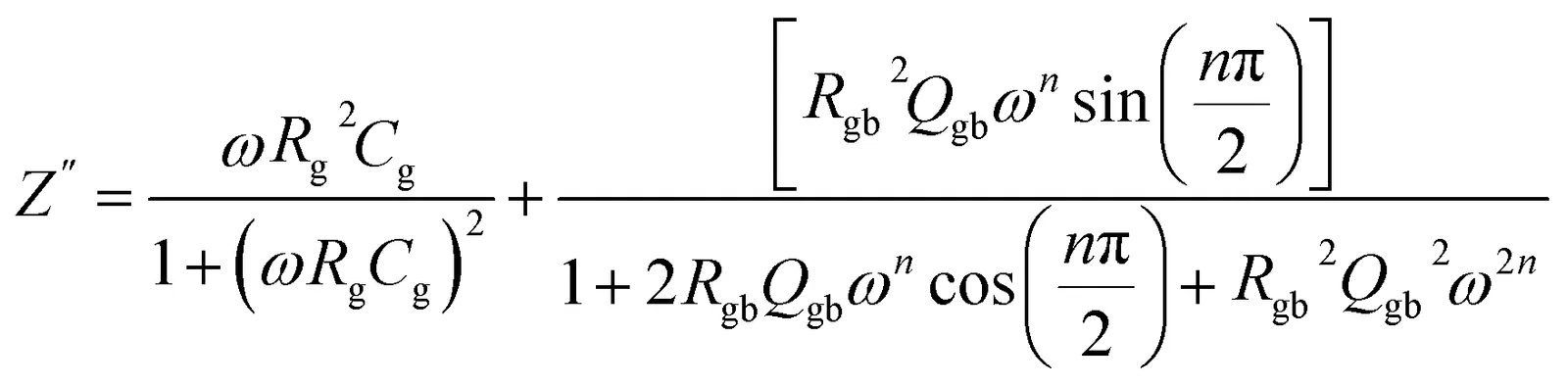


The non-Debye-type dielectric relaxation behavior is indicated by the partially depressed semicircular arcs seen in the Nyquist plots of α-quartz-type GeO_2_ in Fig. 7c. The incomplete semicircles are caused by the overlapping contributions from grains and grain boundaries, limited frequency response within the observed range, and dispersed relaxation times. Interfacial polarization effects, defect-induced localized states, oxygen vacancies, and structural disorder are the reasons for the deviations from ideal Debye-like behavior. Additionally, non-ideal capacitive relaxation linked to heterogeneous grain boundary areas and thermally activated hopping conduction mechanisms is suggested by the existence of continuous phase element (CPE) contributions in the grain boundary. The variation of grain resistance (*R*_g_) and grain boundary resistance (*R*_gb_) with temperature is represented in Fig. 7d, obtained from the output parameters of Nyquist fitting. Both grain and grain boundary resistances gradually decrease as the temperature rises, suggesting that GeO_2_ exhibits a thermally activated semiconducting nature and increased charge carrier mobility. The high grain boundary resistance at lower temperatures is due to defect states, oxygen vacancies, and charge carrier trapping. As the temperature rises, charge carriers become more effective in overcoming potential barriers at grain boundaries, enhancing hopping conduction and decreasing impedance. The temperature dependence of grain and grain boundary resistances follows the Arrhenius relation, and their activation energy (*E*_a_) was calculated using,^64,65^29
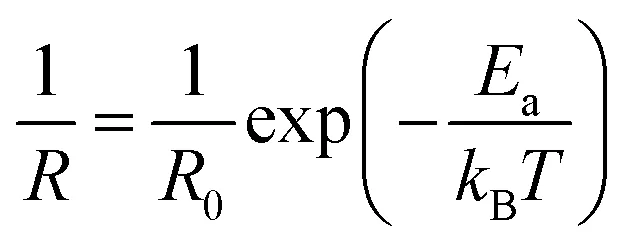


The variations of 
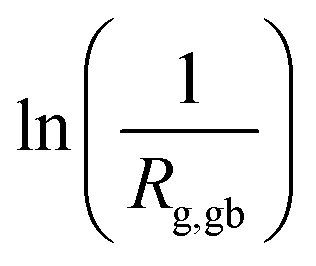
 with 1000/*T* are shown in Fig. 7d-inset, and the *E*_a_ values were obtained as 0.45 eV and 0.78 eV for grain and grain boundary. The grain boundary activation energy is higher than the grain activation energy, suggesting that charge carrier transport across grain boundaries demands greater thermal activation due to defect states, oxygen vacancies, and interfacial trapping effects. The comparison of different activation energies of GeO_2_ obtained from electrical processes is highlighted in Table 1.

**Table 1:** Comparison of different activation energies obtained from various electrical processes

Analysis technique	Arrhenius relation	Physical process	Activation energy (eV)
Grain resistance (*R*_g_) from impedance	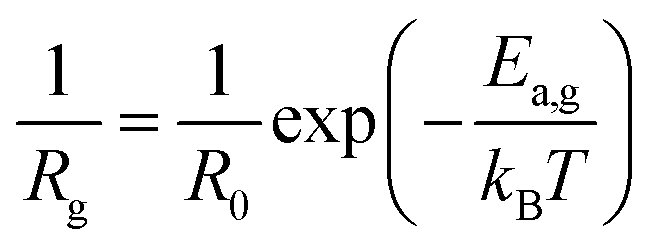	Bulk (grain) conduction	0.45 ± 0.02
Grain-boundary resistance (*R*_gb_) from impedance	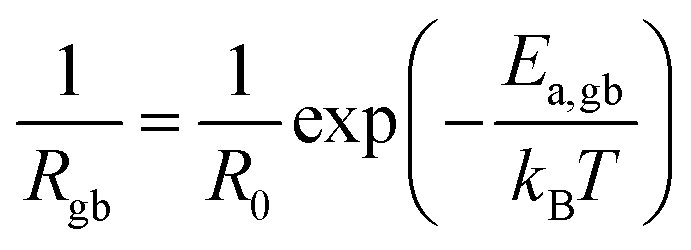	Grain-boundary conduction	0.78 ± 0.03
DC conductivity	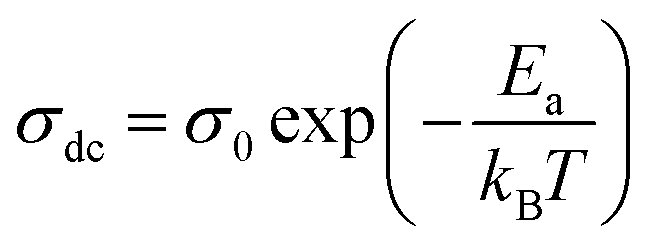	Long-range charge transport	0.98 ± 0.01
Grain resistance (*R*_g_) from modulus	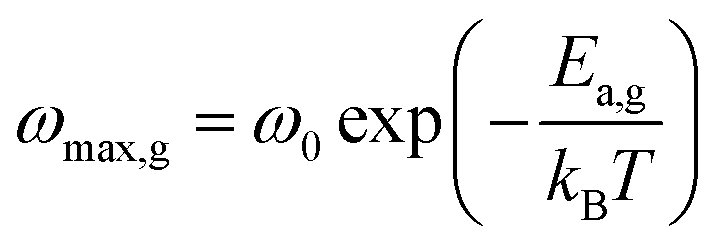	Bulk dielectric relaxation	0.48 ± 0.01
Grain boundary resistance (*R*_gb_) from modulus	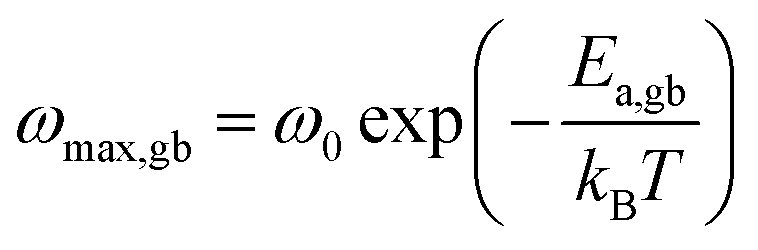	Grain-boundary conduction	0.81 ± 0.02

The study of electric modulus formulism is important because it effectively suppresses electrode polarizations and enhances the ability to distinguish grain and grain-boundary contributions, thereby improving the resolution of relaxation peaks associated with defect-induced polarization mechanisms. The complex modulus (M*) is defined as the reciprocal of complex dielectric permittivity (ε*), and it is expressed as,30
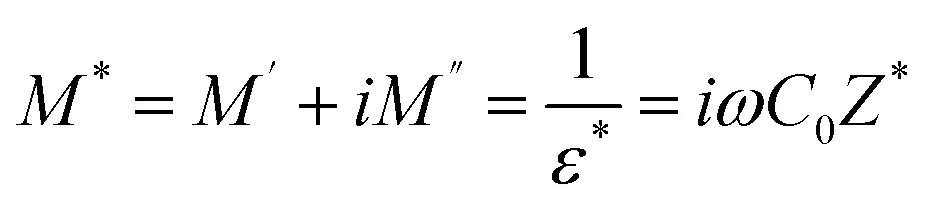
where, 
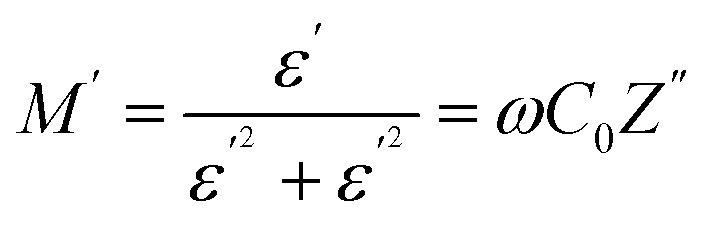
 and, 
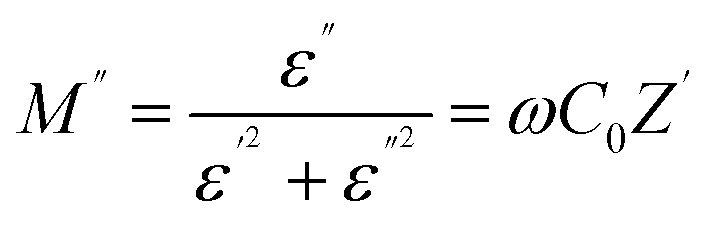
.

**Fig. 8:**
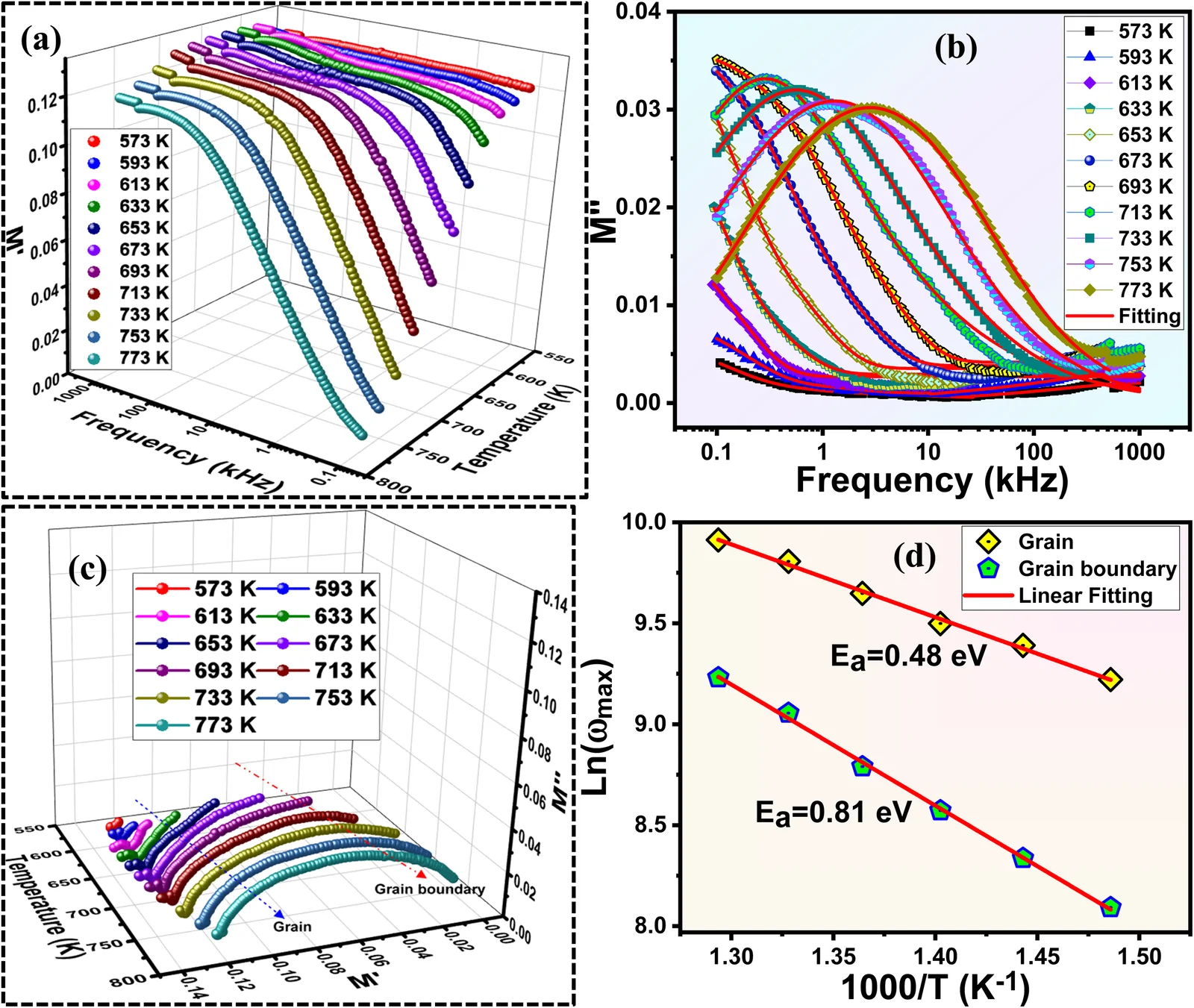
(a) The 3D plot of real modulus (*M*′) with frequency and temperature, (b) the fitted imaginary modulus (*M*″) with frequency at various temperatures. (c) The Cole–Cole plot of modulus (*M*′ *vs. M*″), (d) the Arrhenius plots ln(*ω*_max_) *vs.* 1000/*T* (K) for the activation energy of grain and grain boundary of GeO_2_.

The 3D plot of real modulus (*M*′) with frequency and temperature is illustrated in Fig. 8a for temperatures from 573 K to 773 K. The *M*′ exhibits low values in the low-frequency region at all temperatures, indicating minimal electrode polarization and a weak restoring force on charge carriers. As frequency increases, *M*′ rises due to dipoles and localized charge carriers not keeping pace with the rapid changes of the electric field. The increased dispersion at higher temperatures points to thermally activated hopping conduction and better charge carrier mobility. At high frequencies, *M*′ approaches a saturation plateau reflecting bulk relaxation behavior. The continuous dispersion and lack of distinct relaxation peaks suggest non-Debye-type dielectric relaxation linked to oxygen vacancies, defect states, and grain boundary effects in the GeO_2_ sample. The variations of *M*″ with frequency are delineated in Fig. 8b for various temperatures, and it exhibits asymmetric relaxation peaks at high temperatures after 713 K, which shift toward higher frequencies with increasing temperature. The grain and grain boundary relaxation processes coexist in GeO_2_ within the observed temperature ranges, resulting in overlapping relaxation peaks. To distinguish between various relaxation contributions, the double Bergman fitting function is used to evaluate the imaginary modulus spectra in Fig. 8b by,^45,52,66^31
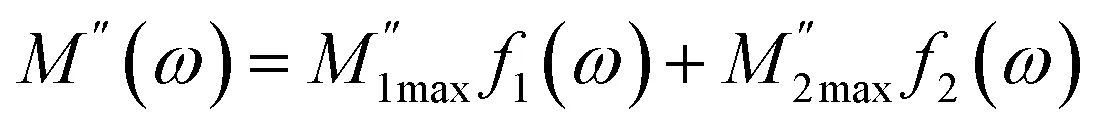
where each *f*_*i*_(*ω*) is,32
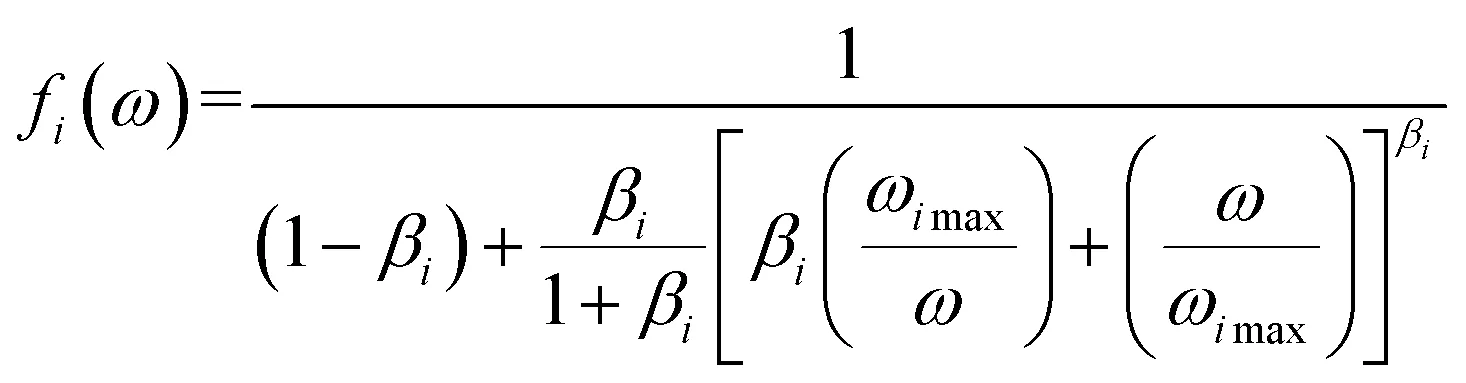
where 
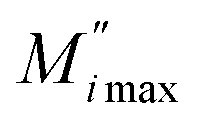
, *ω*_*i*max_, and *β*_*i*_ are the peak maxima of *M*″, peak intensities, and stretching components (0 < *β* ≤ 1) for grain and grain boundary, respectively. The non-Debye relaxation process and localized hopping conduction within the α-quartz-type GeO_2_ sample are confirmed by the obtained stretching parameter (*β* < 1). The Cole–Cole plots of electric modulus spectra (*M*′ *vs. M*″) in Fig. 8c exhibit depressed and asymmetric semicircular arcs, indicating the combined grain and grain boundary contributions and non-Debye-type dielectric relaxation with a distribution of relaxation times. The relaxation peak frequencies (*f*_max_) derived from the fitting of imaginary modulus (*M*″) plots were used to calculate the activation energies (*E*_a_) of grain and grain boundary relaxation of GeO_2_. The *E*_a_ values were determined from the slope of the Arrhenius equation,^67,68^33
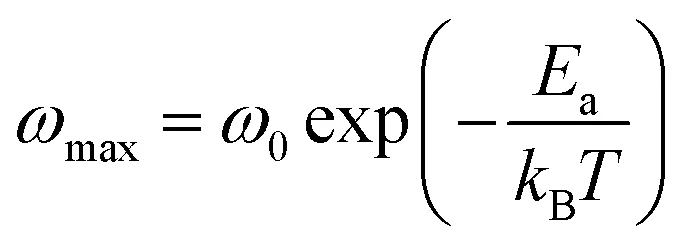
and the obtained *E*_a_ for grain and grain boundary in Fig. 8d were 0.48 eV and 0.81 eV, respectively. These values are well-correlated with the activation energy obtained from the impedance analysis, and it indicates that the same charge carriers are involved in both conduction and relaxation processes. The comprehensive statistical analysis of the Cole–Cole, modified Jonscher power law, impedance spectra & Nyquist plots, and electric modulus non-linear fittings is shown in Table 2.

**Table 2:** The values of statistical analysis of all non-linear fittings related to electrical characterization

Model	Coefficient of determination (*R*^2^)	Adjusted *R*^2^	Reduced chi-square (*χ*^2^)	Root mean square error (RMSE)	Parameter uncertainty
Cole–Cole dielectric fitting	0.979–0.996	0.9924	6.4 × 10^−5^	4.65 × 10^−4^	<3.5%
Modified Jonscher model fitting	0.934–0.986	0.9963	5.3 × 10^−4^	9.5 × 10^−3^	<2.3%
Impedance and Nyquist plots (equivalent circuit)	0.958–0.985	0.9927	2.5 × 10^−5^	3.6 × 10^−4^	<4.8%
Electric modulus fitting	0.968–0.996	0.9986	7.7 × 10^−4^	7.2 × 10^−3^	<3.3%

**Fig. 9:**
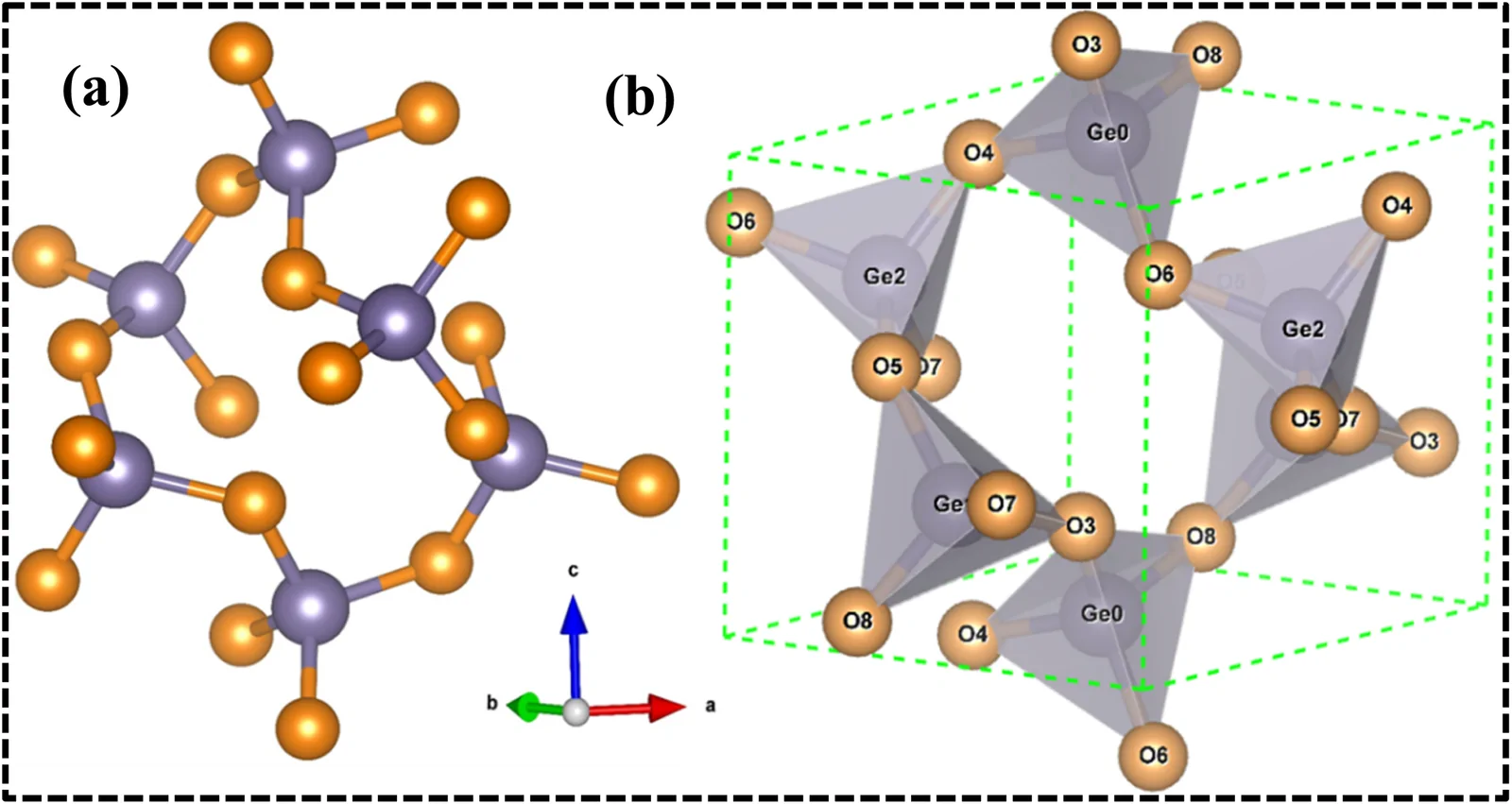
DFT-optimized relaxed structure of bulk GeO_2_ in (a) ball & stick, (b) polyhedral form.

The optimized unit cell structure of α-quartz-type GeO_2_ was examined using DFT to assess its structural stability, electronic and dielectric characteristics, and the optimized structure of GeO_2_ is shown in Fig. 9a and b in ball & stick and polyhedral form. The structure belongs to a trigonal quartz-type crystal system, featuring tetrahedrally coordinated Ge atoms with oxygen, forming interconnected GeO_4_ tetrahedra. The final optimized structure has lattice parameters *a* = *b* = 4.95 Å and *c* = 5.70 Å, which are comparable with the previously reported lattice parameters *a* = *b* = 4.987 Å and *c* = 5.652 Å for GeO_2_.^17,69,70^ The optimized Ge–O bond lengths 1.77 Å and O–Ge–O angles 106.62° support stable tetrahedral coordination and highlight strong covalent interactions between Ge and O, which enhance structural stability and influence electronic properties of the materials.

**Fig. 10:**
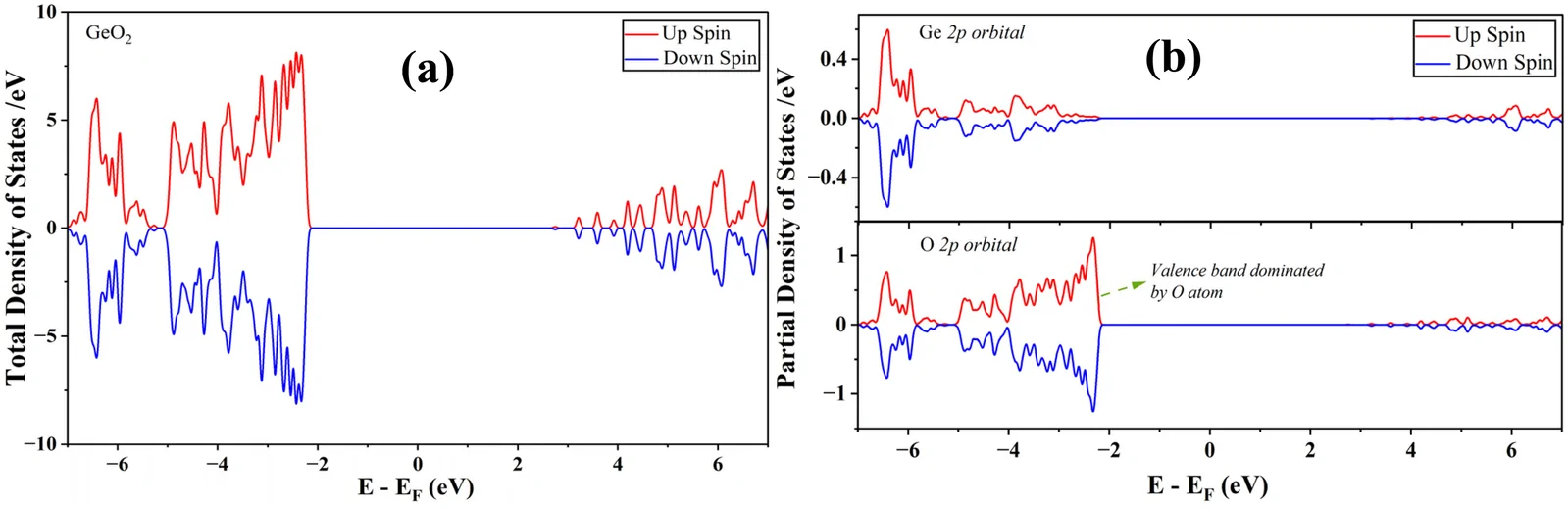
(a) Total density of states (TDOS), (b) partial density of states (PDOS) analysis of Ge and O atoms in GeO_2_.

The total density of states (TDOS) and partial density of states (PDOS) of α-quartz-type GeO_2_ were estimated using the HSE06 approximation to explore its electronic structure, as shown in Fig. 10a and b, respectively. The TDOS reveals a wide-bandgap semiconducting nature of bulk GeO_2_, with a clear separation between the valence band maximum (VBM) and conduction band minimum (CBM), and the bandgap was obtained to be ∼4.59 eV. This value closely matches the value mentioned in the literature.^71,72^ The wide bandgap arises from strong hybridization of Ge and O orbitals in the GeO_4_ framework. The up-spin and down-spin states are exactly symmetric, and the system is non-magnetic, and no spin polarization occurs. The optical band gap observed experimentally differs from the DFT-calculated electronic band gap due to intrinsic and methodological issues. The GGA-PBE functional used in electronic structure calculations typically underestimates band gaps more in ultrawide and wide-bandgap oxides. Additionally, DFT assumes an ideal crystal at 0 K, while experiments on polycrystalline α-quartz GeO_2_ were conducted at room temperature, affected by defects and other factors. DFT predicts the fundamental gap, while experimental results pertain to optical transition energy, possibly influenced by excitonic effects.

According to Fig. 10b, the PDOS analysis shows that the valence band is primarily made up of Ge-2p and O-2p orbital states, emphasizing the importance of oxygen in the electronic structure. It further indicates strong p–p hybridization between the two atoms, confirming the covalent nature of Ge–O bonding. There are no states near the Fermi level, further reinforcing the semiconducting nature of the material, and the states are dominated by the O atom near the Fermi level. Usually, the absence of mid-gap states in defect-free α-GeO_2_ signifies its stable insulating characteristics, but the oxygen vacancies could introduce localized states in the bandgap, affecting dielectric relaxation and charge transport. The phonon calculations can further enhance the understanding of dynamical stability and lattice vibrational properties of α-GeO_2_.

**Fig. 11:**
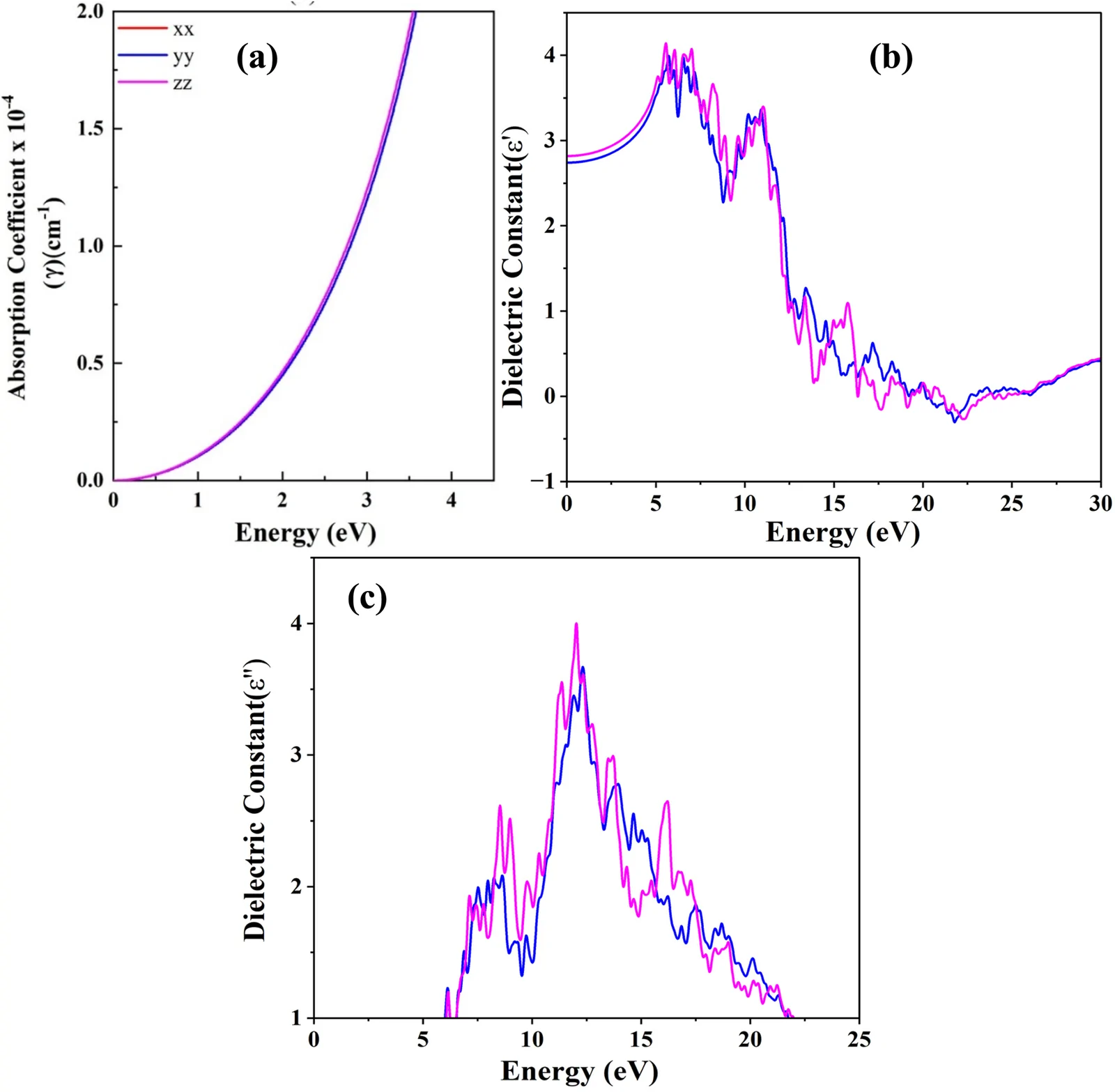
The DFT-calculated (a) absorption coefficient (*γ*), (b) real (*ε*′), and (c) imaginary (*ε*″) parts of the dielectric constant of GeO_2_ as a function of energy (eV).

The optical properties of quartz-type α-GeO_2_ were systematically investigated from DFT results by analyzing the absorption coefficient (*γ*) and complex dielectric function (ε*). These parameters reveal insights into electronic transitions, polarization response, and photon interaction mechanisms in the trigonal crystal structure. The variations of the optical absorption coefficient (*γ*), real (*ε*′) and imaginary (*ε*″) parts of the dielectric function with energy (eV) of GeO_2_ are represented in Fig. 11a–c, respectively. The optical properties of α-GeO_2_ are obtained from the complex dielectric function by ε*(*ω*) = *ε*′(*ω*) + i*ε*″(*ω*), which is related to its electronic structures. The joint density of states and the momentum matrix elements between the occupied and unoccupied wave functions within the selection rules are used to calculate the imaginary part (*ε*″) from the electronic structure, which is provided by,^18,73^34
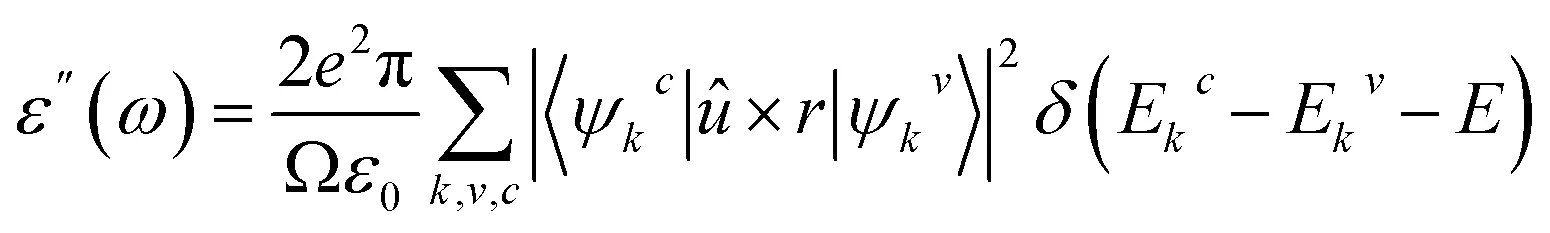
where *Ω* is the unit cell volume, *e* is the electronic charge, and *ψ*_*k*_^*c*^ and *ψ*_*k*_^*v*^ are the conduction band and valence band wavefunctions at the *k*-point (Brillouin zone), respectively. The real part (*ε*′) in Fig. 11b of the dielectric function was calculated using the Kramers–Kronig relationship from *ε*″. The optical absorption coefficient (*γ*) was calculated from both dielectric functions by35
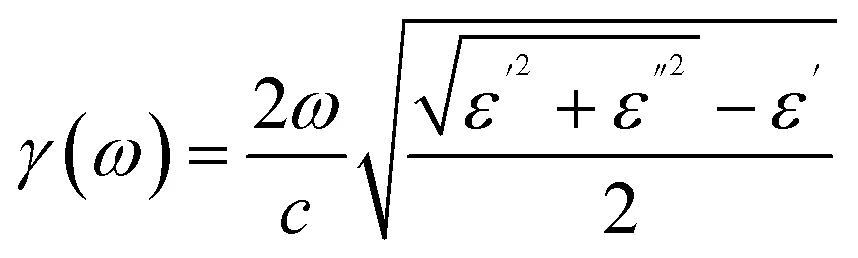
where *c* is the speed of light. Due to the wide bandgap of α-GeO_2_, the computed absorption spectra exhibit very small absorption in the low-energy region, and the interband electronic transitions from O-2p valence states to Ge-2p conduction states are associated with a dramatic increase in the absorption coefficient close to the absorption edge. In the *ε*″ spectrum (Fig. 11c), the broad peaks observed in the mid-energy region between 10 and 15 eV indicate strong optical absorption associated with Ge–O covalent bonding and electronic hybridization within the GeO_4_ tetrahedral network. The *ε*′ spectrum exhibits good electrical polarizations and dispersions under electromagnetic excitation. The trends observed are in good agreement with the previous findings and confirms the reliability of our present study.^8,74,75^

## 4 Conclusions

In summary, α-quartz-type GeO_2_ was successfully synthesized by the solid-state route and systematically investigated for its structural, morphological, electrical, and dielectric properties with theoretical DFT analysis. The Rietveld refinement of XRD, Raman, and FTIR analysis confirmed the formation of highly crystalline GeO_2_ with trigonal crystal symmetry (space group-*P*3_1_21 or *P*3_2_21) and characteristic phonon vibrational modes A_1g_ and E_g_ associated with Ge–O–Ge bonding and GeO_4_ tetrahedral units, and characteristic Ge–O–Ge stretching vibrations, respectively. FESEM analysis showed uniformly distributed micro- and nanostructured grains with good crystallinity, an average size of 538.27 ± 0.23 nm, and strong connectivity; EDX confirmed balanced stoichiometry and composition of the GeO_2_. The Ge-3d and O-1s XPS spectra indicate the near-stoichiometric germanium oxide (GeO_2−*x*_), lattice oxygen, oxygen vacancies, and defect-related states affecting electrical and dielectric properties. UV-vis analysis exhibits an ultrawide bandgap of ∼5.8 eV with strong optical absorption in the ultraviolet range, suggesting its potential for optoelectronic and dielectric applications. High dielectric dispersion (with dielectric constant ∼10.5 and loss *δ* ∼0.03 at 303 K) at low frequency is observed due to space charge and interfacial polarization effects, and a non-Debye Cole–Cole relaxation model is correlated with the observed dielectric response. The AC conductivity was fitted by a modified Jonscher's power law, which allows for a unified analytical description of low-frequency electrode/interfacial polarization, intermediate-frequency relaxation, and high-frequency dispersive conduction, and the frequency exponent with temperature explained the correlated barrier hopping (CBH) model. The decrease in frequency exponent with increasing temperature confirmed defect-assisted hopping conduction mediated by oxygen vacancies and localized defect states, and the dc activation energy was calculated to be 0.98 eV. The impedance spectra, together with complementary modulus spectra, demonstrated thermally activated conduction behavior, significant grain and grain-boundary contributions with activation energies of 0.45 eV and 0.78 eV, and non-Debye-type relaxation behavior. The DOS analysis shows that the valence band is predominantly composed of O-2p states, while the conduction band is mainly composed of Ge-2p states. Additionally, the dielectric functions and absorption coefficient (*γ*) indicate significant ultraviolet absorption and strong dielectric stability of the material. The combined experimental and theoretical investigations recommend the quartz-type α-GeO_2_ as a promising candidate for dielectric, optoelectronic, and high-temperature electronic device applications.

## Conflicts of interest

The authors have no conflicts or competing financial interests to disclose.

## Data Availability

The data that support the findings in this study are available within the article.
